# Two Distinct Conformations in 34 FliF Subunits Generate Three Different Symmetries within the Flagellar MS-Ring

**DOI:** 10.1128/mBio.03199-20

**Published:** 2021-03-02

**Authors:** Norihiro Takekawa, Akihiro Kawamoto, Mayuko Sakuma, Takayuki Kato, Seiji Kojima, Miki Kinoshita, Tohru Minamino, Keiichi Namba, Michio Homma, Katsumi Imada

**Affiliations:** a Department of Macromolecular Science, Graduate School of Science, Osaka University, Toyonaka, Osaka, Japan; b Graduate School of Frontier Biosciences, Osaka University, Suita, Osaka, Japan; c Institute for Protein Research, Osaka University, Suita, Osaka, Japan; d Division of Biological Science, Graduate School of Science, Nagoya University, Nagoya, Japan; e RIKEN Spring-8 Center and Center for Biosystems Dynamics Research, Suita, Osaka, Japan; f JEOL Yokogushi Research Alliance Laboratories, Osaka University, Suita, Osaka, Japan; University of Utah

**Keywords:** bacterial flagellar motor, rotor, MS-ring, type III secretion

## Abstract

The bacterial flagellum is a protein nanomachine essential for bacterial motility. The flagellar basal body contains several ring structures. The MS-ring is embedded in the cytoplasmic membrane and is formed at the earliest stage of flagellar formation to serve as the base for flagellar assembly as well as a housing for the flagellar protein export gate complex. The MS-ring is formed by FliF, which has two transmembrane helices and a large periplasmic region. A recent electron cryomicroscopy (cryoEM) study of the MS-ring formed by overexpressed FliF revealed a symmetry mismatch between the S-ring and inner part of the M-ring. However, the actual symmetry relation in the native MS-ring and positions of missing domains remain obscure. Here, we show the structure of the M-ring by combining cryoEM and X-ray crystallography. The crystal structure of the N-terminal half of the periplasmic region of FliF showed that it consists of two domains (D1 and D2) resembling PrgK D1/PrgH D2 and PrgK D2/PrgH D3 of the injectisome. CryoEM analysis revealed that the inner part of the M-ring shows a gear wheel-like density with the inner ring of C23 symmetry surrounded by cogs with C11 symmetry, to which 34 copies of FliF_D1–D2_ fitted well. We propose that FliF_D1–D2_ adopts two distinct orientations in the M-ring relative to the rest of FliF, with 23 chains forming the wheel and 11 chains forming the cogs, and the 34 chains come together to form the S-ring with C34 symmetry for multiple functions of the MS-ring.

## Abstract (precis)

The bacterial flagellum is a motility organelle formed by tens of thousands of protein molecules. At the earliest stage of flagellar assembly, a transmembrane protein, FliF, forms the MS-ring in the cytoplasmic membrane as the base for flagellar assembly.

## INTRODUCTION

**FIG 1:**
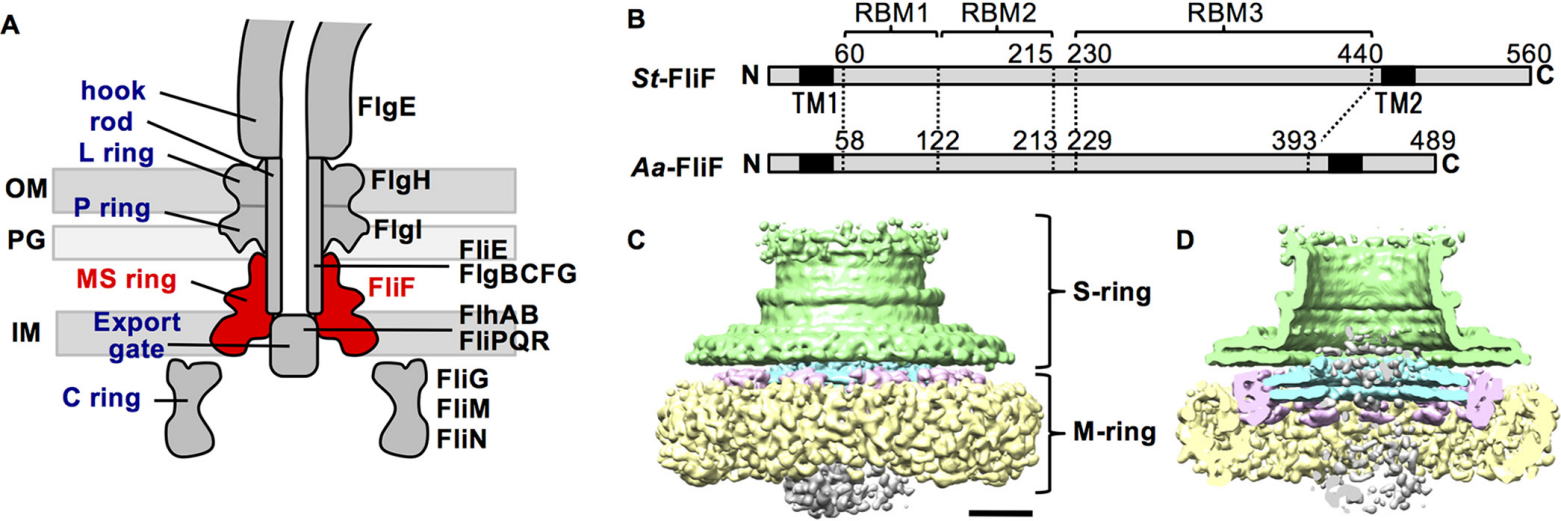
CryoEM density map of the MS-ring. (A) Schematic diagram of the flagellar motor of the Gram-negative bacteria. The name of the structural parts and the proteins are shown on left and right, respectively. The MS-ring (FliF) is colored in red. IM, inner membrane; PG, peptidoglycan layer; OM, outer membrane. (B) Schematic representation of the primary structure of FliF. *St*-FliF and *Aa*-FliF are FliF from *Salmonella* sp. and *A. aeolicus*, respectively. TM, transmembrane; RBM, ring building motif. (C) Surface representation of the cryoEM map of the purified MS-ring at 8.6-Å resolution. Green, the S-ring; cyan, the inner ring of the M-ring; pink, the middle region of the M-ring; yellow, the unstructured outermost region of the M-ring; gray, the unstructured innermost plug region. The scale bar is 50 nm. (D) Vertical section of C.

The bacterial flagellum is a filamentous organelle for locomotion in many bacterial species. The flagellar filament is rotated by a motor embedded in the cell membrane and functions as a screw to thrust the cell. The motor consists of the rotor and several stator units (1, 2). The stator unit is a transmembrane complex of the MotA and MotB family proteins. The rotor is composed of the MS-ring and the C-ring (Fig. 1A). The MS-ring is a transmembrane ring assembly of a single protein, FliF, and has a two-tier ring structure; the S-ring is an upper ring located in the periplasm, and the M-ring is a lower ring embedded in the inner membrane (3). FliF is a 60-kDa protein with a large periplasmic region between two transmembrane helices (Fig. 1B) (4). The C-ring is a cytoplasmic cup-like structure composed of FliG, FliM, and FliN proteins. The torque is generated by the interaction between FliG and MotA and is transmitted from the rotor to the flagellar filament through a drive shaft called the rod, followed by a universal joint called the hook (Fig. 1A).

The MS-ring provides a base for the assembly of the flagellar structure. The C-ring is attached to the cytoplasmic surface of the MS-ring through the interaction between FliG and FliF (5–7). The rod construction begins in the central hole of the MS-ring at its periplasmic side, and thus, the proximal end of the rod is inserted in the MS-ring. The filamentous part of the flagellum, including the rod, hook, and filament, is termed the flagellar axial structure. The component proteins of the axial structure are translocated across the inner membrane via the flagellar protein export apparatus, which is a member of the type III secretion (T3S) family (8, 9). The MS-ring holds a transmembrane region of the export apparatus in its central hole and therefore serves as housing for the export apparatus (Fig. 1A). The flagellar formation is thought to be initiated by the assembly of the type III export gate complex composed of FlhA, FlhB, FliP, FliQ, and FliR with the help of the FliO scaffold (10), followed by the recruitment of FliF through an interaction between FliF and FlhA (11) to form the MS-ring around the export gate complex (10). The export gate component proteins form partial gate complexes but cannot assemble into the complete export gate complex without FliF, indicating that the MS-ring stabilizes the export gate complex (10, 12).

The proteins of the flagellar basal body structure are homologous to the component proteins of the T3S injectisome, a needle-like organelle of pathogenic bacteria that delivers effector proteins into host cells for infection and pathogenicity (13). The flagellar basal body proteins also show similarities to the component proteins of the sporulation-essential channel (14, 15), which connects the mother cell and the forespore in Bacillus subtilis. FliF shares homology with the inner membrane ring components of the T3S injectisome SctJ (also named as EscJ in enteropathogenic Escherichia coli [EPEC] and PrgK in *Salmonella* SPI-1) and SctD (EscD in EPEC and PrgH in SPI-1) and the *Bacillus* sporulation channel components SpoIIIAH and SpoIIIAG (14–18). Previous sequence analysis revealed that the periplasmic region of FliF has three ring-building motifs (RBMs) (15), namely, RBM1, RBM2, and RBM3 (Fig. 1B), which are conserved in SctJ/SctD, SctJ/SctD/SpoIIIAH, and SpoIIIAG, respectively. X-ray crystallography and electron cryomicroscopy (cryoEM) analyses of the *Salmonella* SPI-I injectisome have revealed that PrgK and PrgH form a concentric double ring with 24-fold rotational symmetry in the periplasmic region of the basal body (18–21). The crystal structures of SpoIIIAF and SpoIIIAH have shown a structural similarity to PrgK (16, 17, 22). The high-resolution cryoEM structure of SpoIIIAG has revealed that SpoIIIAG forms a 30-fold symmetrical ring with a unique cylindrical β-barrel structure (23).

Recently, partial structures of the periplasmic region of the MS-ring have been determined at 2.6- to 3.3-Å resolution by cryoEM image analysis of purified MS-rings formed by recombinant FliF with some C-terminal truncations. (24). These structures showed a variation in their subunit stoichiometry and revealed that the S-ring shows C32 to C35 symmetry and consists of the C-terminal half of the periplasmic region of FliF, including RBM3. The S-ring comprises a globular domain forming a flat ring and an extended chain, including long antiparallel β-strands forming a cylindrical collar above the ring. The overall S-ring structure is similar to that of SpoIIIAG, albeit their symmetries are different. In addition, 21 or 22 copies of RBM2 form a ring in the inner part of the M-ring, and this is surrounded by 9 or 10 globular densities composed of RBM1 and RBM2. However, a few copies of RBM2 and more than 20 copies of RBM1 are missing in these structures, and the variation in the subunit stoichiometry is likely to be an artifact due to C-terminal truncations of FliF because the MS-ring in the flagellar basal body, as well as that formed by full-length recombinant FliF, showed only 34-fold rotational symmetry (25). Moreover, the cryoEM analysis of the purified the flagellar basal body showed a C23 symmetry at the inner part of the M-ring (25). Therefore, the roles and functions of the RBM domains in the MS-ring formation and entire flagellar assembly remain obscure.

Here, we report the crystal structure of *Aa*-FliF_58–213_, which corresponds to the N-terminal half of the periplasmic region of FliF from Aquifex aeolicus, at 2.3-Å resolution. FliF_58–213_ is composed of two domains (D1 and D2), and they show structural similarity to the corresponding domains of SctJ, SctD, and SpoIIIA. We constructed an atomic model for the inner part of the M-ring by combining the crystal structure with low-resolution cryoEM maps of the MS-ring with the help of structural similarity to the homologous injectisome proteins. The model indicates that FliF subunits adopt two distinct conformations in the M-ring structure to generate multiple symmetries within the ring. We also built a structural model of the entire periplasmic region of FliF by combining with the S-ring model determined by high-resolution cryoEM analysis (25). These results provide the structural basis of the flagellar assembly mechanism and the evolutionary relation to the T3S injectisome and sporulation channel.

## RESULTS

### CryoEM analysis of the *Salmonella* MS-ring complex.

**FIG 2:**
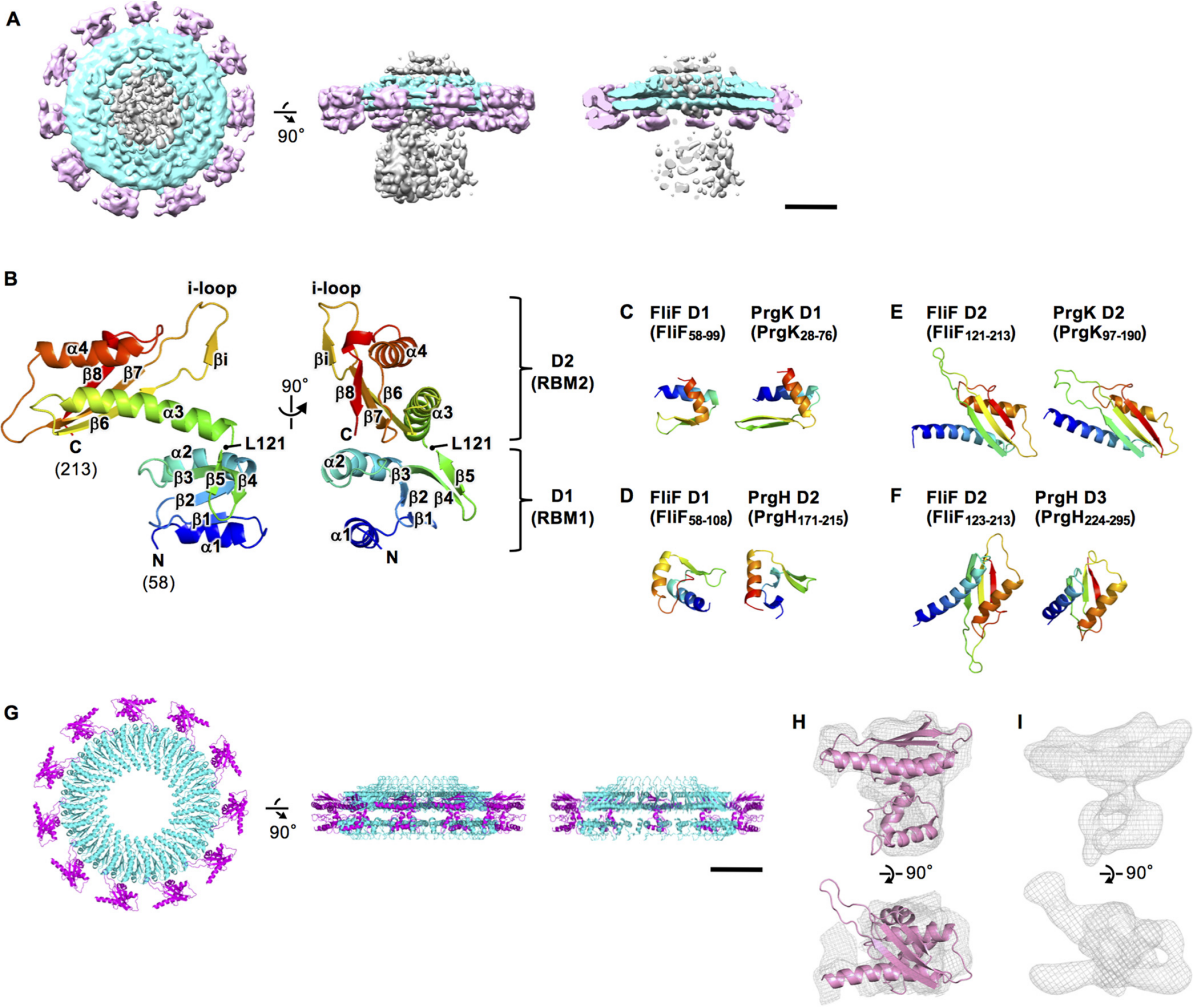
Structure of RBM1 and RBM2 of FliF. (A) The cryoEM surface map of the inner ring and the middle region of the M-ring with the central blurred density. The top view, the side view, and the vertical section are shown in the left, the middle, and the right, respectively. The scale bar is 50 nm. (B) Cα ribbon drawing of the crystal structure of *Aa*-FliF_58–213_. The model is color coded from blue to red from the N to the C terminus. The structure consists of two domains, namely, D1 (residues 58 to 122) and D2 (residues 123 to 213). (C to F) Comparison of the domain structures of *Aa*-FliF with those of PrgH and PrgK. (G) The structure model of the inner ring (cyan) and the middle region (magenta) of the M-ring. The top view, the side view, and the vertical section are shown in the left, the middle, and the right, respectively. (H) Superimposition of the D1 to D2 structure (pink) on the cryoEM map of the middle region of the M-ring after applying the 11-fold symmetry averaging (gray mesh). (I) 8.6-Å density maps of the middle region calculated from the D1 to D2 structure model.

We expressed and purified the *Salmonella* MS-ring with FliG for single-particle cryoEM image analysis (see Fig. S1 in the supplemental material). Reconstruction of the MS-FliG ring complex without imposing any symmetries yielded an 8.6-Å resolution map (Fig. 1C and D). The map showed that the purified MS-ring consists of the following five regions: (i) the S-ring, (ii) the inner ring of the M-ring, (iii) the middle region of the M-ring with C11 symmetry, (iv) the outermost region of the M-ring, and (v) the innermost plug region of the M-ring (Fig. 1C and D, Fig. 2A). The maps of the outermost region and the innermost plug region of the M-ring show a blurred density, which may include the transmembrane region of FliF with detergent molecules. The overall view of the map is similar to that of the MS-ring map reported recently (24), although the rotational symmetry of each region is different. Unfortunately, the density corresponding to the FliG ring was invisible.

10.1128/mBio.03199-20.1 FIG S1 3D reconstruction of the MS-ring. (A) Three-dimensional classification scheme. (B) Selected 2D class averages. (C) The FSC curves of the C1 and C11 reconstructions of the MS-ring. Download FIG S1, TIF file, 1.8 MB. Copyright © 2021 Takekawa et al. 2021 Takekawa et al. https://creativecommons.org/licenses/by/4.0/ This content is distributed under the terms of the Creative Commons Attribution 4.0 International license.

### Crystal structure of a FliF fragment corresponding to RBM1 and RBM2.

Partial atomic models of the MS-ring corresponding to the S-ring (RBM3) and the inner part of the M-ring (RBM2_inner_) have already been determined based on high-resolution cryoEM maps (24). The middle part of the M-ring (RBM2_outer_) has also been modeled using medium-resolution cryoEM maps (24). However, RBM1 and a few copies of RBM2 remain unknown and are required for understanding their roles in the M-ring function. To build the atomic model of RBM1 and RBM2, we determined the crystal structure of a FliF fragment containing these two motifs. We initially tried to crystallize various fragments of *Salmonella* FliF, but no crystal was obtained. Therefore, we prepared FliF fragments from a thermophilic bacterium, *A. aeolicus*, and determined the crystal structure of a fragment consisting of residues 58 to 213 (*Aa*-FliF_58–213_) at 2.3-Å resolution (Fig. 2B; see Fig. S2A to E in the supplemental material; see Table S1 in the supplemental material). The crystal belongs to the space group of *H*3 and contains two essentially identical molecules in an asymmetric unit (Fig. S2E) (the root mean square deviation for Cα atoms is 0.694). The structure of *Aa*-FliF_58–213_ consists of two distinct domains corresponding to RBM1 (D1; Pro58 to Ser122) and RBM2 (D2; Arg123 to Asp213) (Fig. 1B, Fig. 2B). The D1 domain (FliF_D1_) is a compact globular domain composed of two α-helices (α1 and α2) and five β-strands (β1 to β5). β1, β2, and β3 form a core β sheet flanked by the two α-helices and another β-sheet made up of β4 and β5. The D2 domain (FliF_D2_) is more elongated and consists of two α-helices (α3 and α4) and four β-strands (β6 to β8 and βi). An intramolecular disulfide bridge is formed between C147 and C182 at a pole of FliF_D2_ but is not essential for the FliF function, as discussed later. *Aa*-FliF_58–213_ forms a dimer related by a pseudo-2-fold symmetry in the crystal, and β4 and β5 form an intersubunit β sheet with βi of the dimer mate (Fig. S2E and F). However, the dimer is a crystal-packing artifact because FliF forms a ring structure, and the 2-fold symmetrical dimer cannot be fitted in the cryoEM density, as shown later. The overall structure of FliF_D2_ is similar to the recently reported cryoEM structures of RBM2_inner_ and RBM2_outer_ (24) (Fig. S2G).

10.1128/mBio.03199-20.2 FIG S2 Construction, purification, and crystallization of FliF fragments. (A) Schematic representation of the primary structures of *A. aeolicus* FliF (*Aa*-FliF) and its fragments used in this study. (B) Expression of *Aa*-FliF and its fragments in E. coli cells examined by SDS-PAGE. 1, whole-cell lysate; 2, cell debris and inclusion body; 3, membrane proteins and macromolecular complexes; 4, soluble fraction. (C) The elution profile of FliF_58-213_ on the size exclusion column (Superdex 75 10/300 GL). The SDS-PAGE result of the peak fraction is shown in the right. (D) Crystals of native FliF_58-213_ (left) and the Se-Met FliF_58-213_ (L121M and L195M) (right). (E) A FliF_58-213_ dimer (cyan and magenta) in the asymmetrical unit of the crystal. (F) Close-up view of the region shown by black box in E. The β-strands (β5 and βi) that form the intersubunit β-sheet in the crystal are indicated by stick model. (G) Structural comparison of RBM2s. RBM2_inner_, RBM2_outer_ (PDB ID: 6SCN), and FliF_D2_ in the crystal structure are shown. Download FIG S2, TIF file, 2.8 MB. Copyright © 2021 Takekawa et al. 2021 Takekawa et al. https://creativecommons.org/licenses/by/4.0/ This content is distributed under the terms of the Creative Commons Attribution 4.0 International license.

10.1128/mBio.03199-20.8TABLE S1Summary of the X-ray data statistics. The values in parentheses are for the highest-resolution shell.
${\text{R}}_{\text{w}}=\Sigma \left|\left|\text{ Fo }\right|-\right|\text{ Fc }\left|\left|/\Sigma \right|\text{ Fo }\right|,{\text{ R}}_{\text{free}}=\Sigma \left|\left|\text{ Fo }\right|-\right|\text{ Fc }\left|\left|/\Sigma \right|\text{ Fo }\right|$. Download Table S1, DOCX file, 0.03 MB.Copyright © 2021 Takekawa et al.2021Takekawa et al.https://creativecommons.org/licenses/by/4.0/This content is distributed under the terms of the Creative Commons Attribution 4.0 International license.

### FliF_D1_ and FliF_D2_ structurally resemble the components of the T3S injectisome.

Amino acid sequence analysis showed that FliF has structural motifs conserved in SctD (PrgK in SPI-1) and SctJ (PrgH in SPI-1) of the T3S injectisome (15). The periplasmic region of PrgK is composed of two domains, namely, D1 (PrgK_D1_) and D2 (PrgK_D2_), and that of PrgH is composed of three domains, namely, D2 (PrgH_D2_), D3 (PrgH_D3_), and D4 (PrgH_D4_) (see Fig. S3A and B in the supplemental material). They form a concentric double ring with C24 symmetry (the inner PrgK ring and the outer PrgH ring) (Fig. S3C). We found that FliF_D1_ shows structural similarity to both PrgK_D1_ and PrgH_D2_ (Fig. 2C and D, Fig. S3D), although the N-terminal α-helix of FliF_D1_ (α1) is longer than the corresponding helix of PrgH_D2_. FliF_D2_ resembles both PrgK_D2_ and PrgH_D3_, which adopt a common RBM fold (Fig. 2E and F, Fig. S3D). These structural similarities suggest that the D1-D2 region of FliF (FliF_D1–D2_) also forms a concentric double-ring structure in a way similar to the PrgK-PrgH ring of the T3S injectisome.

10.1128/mBio.03199-20.3 FIG S3 Construction of the atomic models of the inner ring and the middle region of the M-ring. (A) The cryoEM structure of the T3S injectisome (EMDB ID: 8544) (41) with the atomic models of the periplasmic ring of PrgH and PrgK (PDB ID: 5TCP) (21). (B) Close-up view in the blue dashed box in A. (C) The top view of the periplasmic ring model in A. (D) Superimposition of FliF_D1–D2_ to the periplasmic region of PrgH (green) and PrgK (orange). FliF_D1–D2_ is colored in cyan and magenta. See also Fig. 2C to F. (E) Domain architecture of *Aa*-FliF, PrgH, and PrgK. The gray boxes indicate the putative transmembrane regions. The blue and red boxes indicate the regions that show structural similarity. (F) The initial ring model of FliF_D1–D2_ based on the structural similarity to the periplasmic ring of PrgH, and PrgK. The model contains 48 sets of FliF_D1–D2_ (inner 24 sets and outer 24 sets). (G) The second ring model of FliF_D1–D2_. The initial model was modified by applying the symmetry information from the cryoEM density map. The model contains 34 sets of FliF_D1–D2_ (inner 23 sets and 11 outer sets). (H) The final ring model of FliF_D1–D2_. The outer 11 subunits were modified as in J and K. (I) The cryoEM density map after applying the 11-fold averaging to the middle part of M-ring. (J) Strategy for the fitting of FliF_D1–D2_ into the averaged cryoEM map of the middle part of the M-ring. FliF_D2_ was rotated against FliF_D1_ to fit into the map. (K) Comparison of the atomic model (top) with the cryoEM map (bottom) corresponding to the single subunit of the middle part of M-ring in various views. Download FIG S3, TIF file, 2.6 MB. Copyright © 2021 Takekawa et al. 2021 Takekawa et al. https://creativecommons.org/licenses/by/4.0/ This content is distributed under the terms of the Creative Commons Attribution 4.0 International license.

### Structure of the inner ring and the middle region of the M-ring.

*Aa*-FliF_58–213_ shows an amino acid sequence identity of 44% with the corresponding region of *Salmonella* FliF (*St*-FliF_60–215_) without any insertions and deletions (see Fig. S4A in the supplemental material). Thus, we made a homology model of *St*-FliF_60–215_ based on the structure of *Aa*-FliF_58–213_ and used this model to construct the M-ring structure. We first built a tentative FliF ring model by superimposing FliF_D1_ and FliF_D2_ to the PrgK and PrgH rings independently (Fig. S3D to F). The tentative ring model contains a total of 48 FliF_D1–D2_ subunits, as follows: 24 subunits form the inner ring (fitted to the PrgK ring), and the remaining 24 subunits form the surrounding ring (fitted to the PrgH ring). The inner and outer diameters of the inner ring of the 24-mer model are comparable to those of the cryoEM density for the inner M-ring with C23 symmetry in the native *Salmonella* basal body (25), suggesting that the tentative 24-mer model has a similar subunit arrangement to the inner M-ring. Therefore, we produced a 23-mer model by applying a C23 symmetry operation to a subunit of the 24-mer inner ring model and then fit it into the cryoEM density with C23 symmetry (EMD-30613) (25) (Fig. S3G). The domain arrangement is similar to those in the recently reported RBM2_inner_ ring models (24), although their symmetries are different, namely, C21 and C22. To further examine the correctness of the subunit arrangement in the inner ring, we replaced some residues of FliF at the subunit interface with cysteine and examined oligomerization by SDS-PAGE, followed by immunoblotting (see Fig. S5 in the supplemental material). The H156C/S200C mutant produced higher-order oligomers by intermolecular disulfide bonds, and this result supports the subunit arrangement in the inner ring model because H156 and S200 are close enough to form a disulfide bond in the ring model.

10.1128/mBio.03199-20.4 FIG S4 Functional analysis of *A. aeolicus* FliF in *Salmonella*. (A) The amino acid sequence alignment of FliF from *Salmonella* (*St*-FliF) and *A. aeolicus* (*Aa*-FliF). The identical and homologous amino acid residues are indicated in black and gray boxes, respectively. The putative transmembrane regions predicted by TMHMM Server version 2.0 (http://www.cbs.dtu.dk/services/TMHMM/) are shown in red boxes. The secondary structures determined by the X-ray crystallography or the cryoEM analysis are colored in blue, and those predicted by PSIPRED are in black. α-Helices and β-strands are indicated by cylinders and arrows, respectively. (B) The architecture of the chimeric FliF. The residues 58 to 214 of *St-*FliF is replaced by the residues 56 to 212 of *Aa-*FliF. (C) Swimming motility of the cells in the soft agar plate. The plates were incubated at 30°C for 6 h for the left and 24 h for the right. The chimeric FliF is functional in *Salmonella* Δ*fliF* cells. (D) Fluorescent analysis of FliF-GFP in the cell. The chimeric FliF-GFP showed fluorescent dots in the cell as with the wild-type *St*-FliF-GFP, suggesting that chimeric FliF can form the MS-ring. (E) The disulfide bridge between C147 and C182 in FliF_D2_. (F) The effect of the disruption of the S-S bond on motility. The plate was incubated at 30°C for 24 h. The C147A mutation abolished the motility but the C182A mutation did not. Download FIG S4, TIF file, 2.5 MB. Copyright © 2021 Takekawa et al. 2021 Takekawa et al. https://creativecommons.org/licenses/by/4.0/ This content is distributed under the terms of the Creative Commons Attribution 4.0 International license.

10.1128/mBio.03199-20.5 FIG S5 Disulfide crosslinking of the putative subunit interface of the inner ring. (A) The structure model of the inner ring and the middle region of the M-ring. Two adjacent D2 domains in the inner ring are colored in green and cyan. (B) Cartoon representation of two adjacent D2 domains in A after energy minimization by using Rosetta Relax application (42). The side chains are shown by stick model. The mutated residues are highlighted in yellow. (C) Detection of disulfide crosslinking products of FliF. The whole-cell lysates of *Salmonella* Δ*fliF* cells expressing wild-type and mutant FliF were analyzed by SDS-PAGE followed by immunoblotting using the anti-FliF antibody. The oligomeric and the monomeric forms of FliF are indicated by black and white arrowheads, respectively. Download FIG S5, TIF file, 0.9 MB. Copyright © 2021 Takekawa et al. 2021 Takekawa et al. https://creativecommons.org/licenses/by/4.0/ This content is distributed under the terms of the Creative Commons Attribution 4.0 International license.

The surrounding ring subunits of the tentative ring model overlapped with the cryoEM density of the middle region of the M-ring, but its symmetry is C11. Thus, we omitted 13 protomers and rearranged the remaining 11 protomers of FliF_D1–D2_ in the tentative surrounding ring by applying C11 symmetry (Fig. S3G). Then, we modified the orientation of each domain by fitting it into the cryoEM density with 11-fold averaging (Fig. 2G and H, Fig. S3H to K). The final model of the inner ring and the middle region shows a gear wheel-like structure containing 34 copies of FliF_D1–D2_, as follows: 23 copies in the inner ring, and 11 copies in the middle region surrounding the inner ring, where 11 copies are equiangularly positioned with a rather large empty space between neighboring copies (Fig. 2G). FliF_D1–D2_ in the middle region and FliF_D2_ in the inner ring are well fitted into the cryoEM density of the MS-ring (Fig. 2H). However, the density corresponding to FliF_D1_ in the inner ring is rather poor (Fig. 2A), maybe because FliF_D1_ of the inner ring is unstable without the export gate complex.

### Functional analysis of a FliF chimera.

To obtain functional evidence that supports our ring model based on the *Aa*-FliF_58–213_ structure, we constructed a FliF chimera protein with residues 58 to 214 of *Salmonella* FliF (*St*-FliF) replaced by residues 56 to 212 of *A. aeolicus* FliF (*Aa-*FliF) (Fig. S4B) and expressed it in *Salmonella* Δ*fliF* cells. The deletion of *fliF* completely abolished motility in soft agar (Fig. S4C) because the flagellum was not produced. Expression of *Aa-*FliF did not complement the motility defect of the *Salmonella* Δ*fliF* mutant, whereas the expression of the FliF chimera restored the motility to a significant degree (Fig. S4C). *Salmonella* Δ*fliF* cells expressing *St*-FliF labeled with green fluorescent protein (GFP) to its C terminus (*St*-FliF-GFP) showed fluorescent dots mostly on the cell surface, suggesting that expressed *St*-FliF-GFP forms the MS-ring (Fig. S4D) (11). Fluorescent dots also appeared on the cell surface of the *Salmonella* Δ*fliF* cells expressing the FliF chimera labeled with GFP, although the number and intensity of the fluorescent dots were less than those of *St*-FliF expressing cells (Fig. S4D), suggesting that the FliF chimera protein can form the MS-ring albeit not at the *St*-FliF level. These results indicate that the structures of *Aa*-FliF_58–213_ and *St*-FliF_60–215_ are similar enough to be exchangeable. We also observed that the C182A mutation, which disrupts the intramolecular disulfide bridge between C147 and C182 in the D2 domain of *Aa*-FliF, did not affect the function of FliF chimera (Fig. S4E and F), indicating that the disulfide bridge is not needed for the MS ring formation in *Salmonella*.

### The “i-loop” in FliF_D2_ is required for assembly of the flagellar export gate complex.

**FIG 3:**
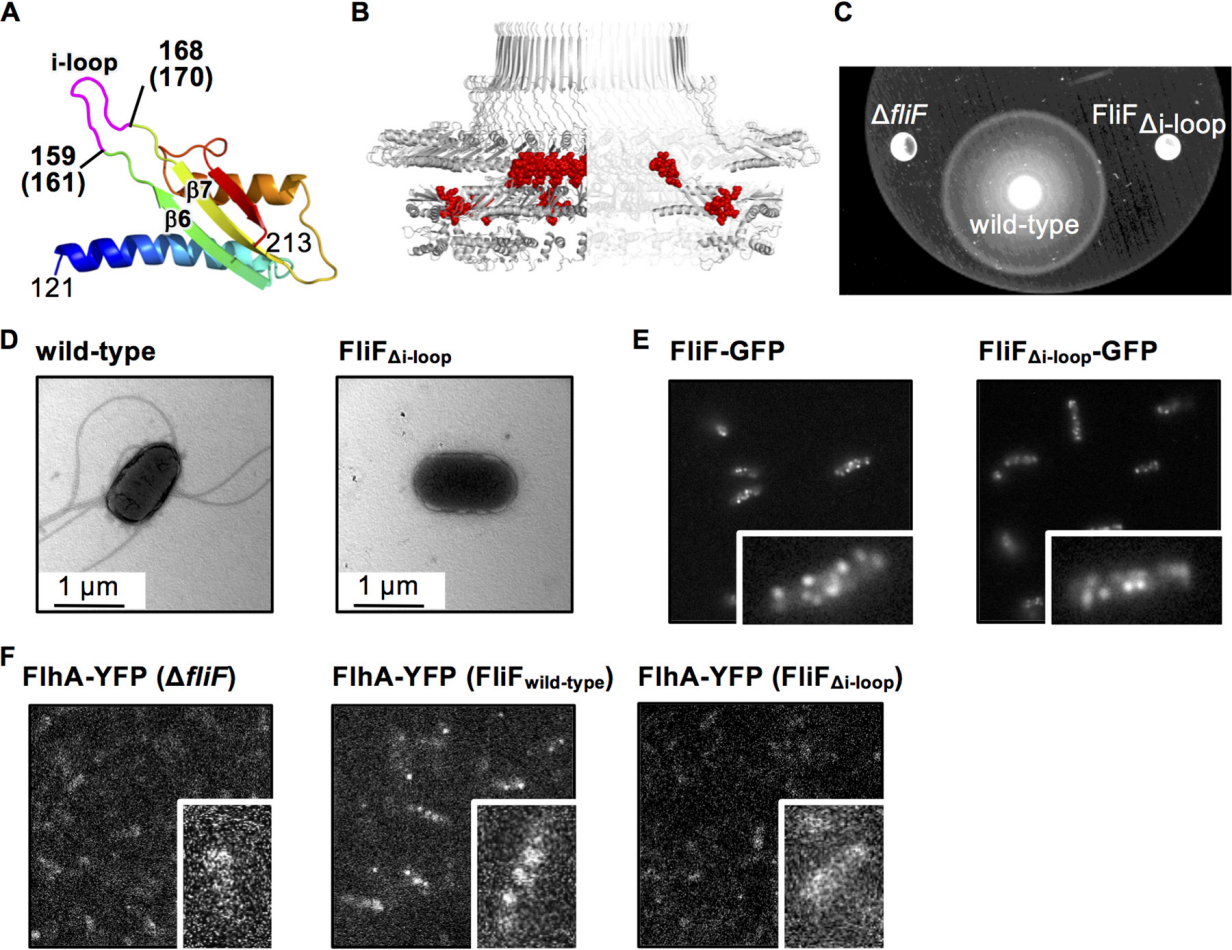
Effect of the deletion of the loop between β4 and β5 (i-loop). (A) Structure of D2 of *Aa*-FliF. The i-loop is highlighted in magenta. The corresponding residue numbers of *St*-FliF are shown in parentheses. (B) The i-loops (red spheres) in the ring model. (C) Swimming motility of *Salmonella* wild-type and FliF_Δi-loop_ mutant cells in the soft agar plate. The plates were incubated at 30°C for 9 h. (D) Electron micrographs of a *Salmonella* wild-type cell and a FliF_Δi-loop_ mutant cell. (E) Subcellular localization of *St*-FliF-GFP and *St*-FliF_Δi-loop_-GFP. (F) Subcellular localization of FlhA-YFP in the *fliF* null mutant, the wild type, and the FliF_Δi-loop_ mutant. Magnified views of single cells are shown in the lower right of each image in E and F.

In our M-ring model, a characteristic protruding loop connecting β6 and β7 and containing βi (residues 157 to 170 in *Aa*-FliF and 159 to 172 in *St*-FliF) (Fig. 3A), termed the i-loop, surrounds the hole of the inner ring (Fig. 3B). To investigate the role of the i-loop in flagellar formation, we deleted residues 161 to 170 of *St*-FliF (FliF_Δi-loop_) to see the phenotype. This deletion completely abolished cell motility and flagellation (Fig. 3C and D). In contrast, fluorescent dots were found in *Salmonella* Δ*fliF* cells expressing FliF_Δi-loop_ labeled with GFP at a similar level to that of the cells expressing wild-type *St*-FliF-GFP (Fig. 3E). The MS-ring is essential for the assembly of the flagellar protein export gate complex composed of FlhA, FlhB, FliP, FliQ, and FliR (11). FlhA forms the homo-nonamer in the complex, and the deletion of *fliF* completely abolishes the assembly of FlhA (11). Because the i-loop is adjacent to A174 and S175 of FliF, whose deletions are partially suppressed by extragenic suppressor mutations in the N-terminal transmembrane domain of FlhA (see Fig. S6 in the supplemental material), we analyzed the effect of i-loop deletion on the assembly of the FlhA ring. FlhA labeled with yellow fluorescent protein (YFP) formed fluorescent dots in the cell expressing wild-type FliF, and the analysis of the fluorescence intensity revealed that FlhA forms nonamer (12) (Fig. 3F), but no clear fluorescent dot was observed in the i-loop deletion mutant (Fig. 3F). These results indicate that the i-loop is important for the FlhA assembly within the MS-ring.

10.1128/mBio.03199-20.6 FIG S6 Mapping of the A174 to S175 deletion mutation site in the ring model and its suppressor mutation sites in FlhA. (A) Ribbon representation of the *Aa*-FliF D2 domain structure. A172 and S173 in *Aa*-FliF, which correspond to A174 and S175 in *St*-FliF, are shown (30). (B) The location of the deletion mutation sites (red spheres) in the ring model. (C) The suppressor mutation sites (M57I, L60Q, L118Q, M138L/C, I148S, G222S, V268L, and S272N) in *Salmonella* FlhA (30). Download FIG S6, TIF file, 0.9 MB. Copyright © 2021 Takekawa et al. 2021 Takekawa et al. https://creativecommons.org/licenses/by/4.0/ This content is distributed under the terms of the Creative Commons Attribution 4.0 International license.

### Structure of the periplasmic region of the MS-ring.

**FIG 4:**
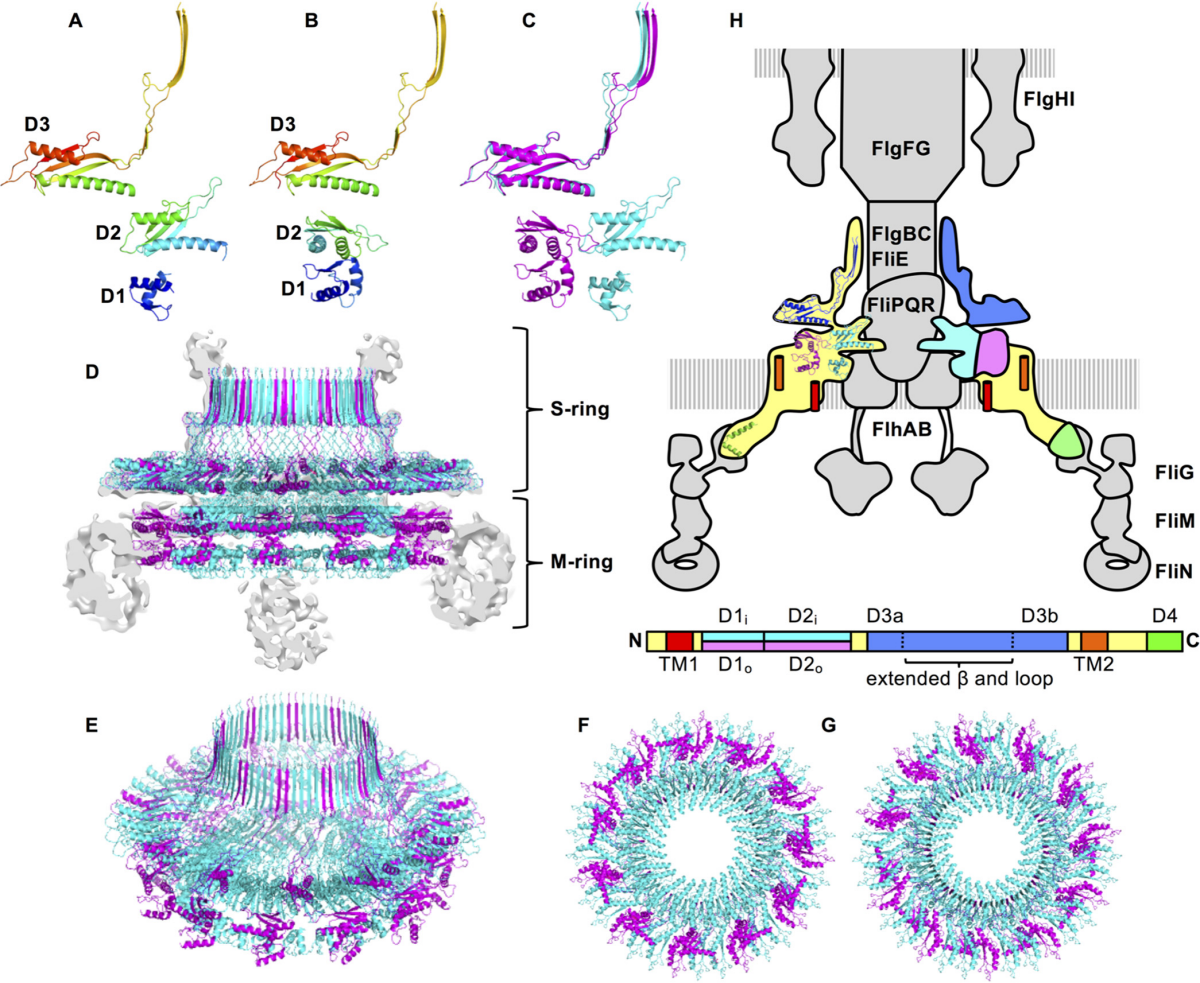
The structure model of the periplasmic region of the MS-ring. (A, B) The two distinct conformations of the FliF subunit in the MS-ring. (A) The structure of the FliF subunit that forms the inner ring (A) and the middle region (B) of the M-ring and the S-ring. (C) Superimposition of A (cyan) and B (magenta). (D to G) Structure of the periplasmic region of the MS-ring. The side view (D), the diagonal view (E), the bottom view (F), and the top view (G) are shown in the same color profile as in C. The gray image in D is the vertical section of the cryoEM density map. (H) Arrangement and location of FliF in the flagellar motor. The MS-ring is colored, and the others are shown in gray in the schematic drawing of the flagellar basal body. The primary structure of FliF with the domain annotation is shown below the basal body. The ribbon model of FliF is overlaid on the left half of the MS-ring. The right half of the MS-ring is colored as in the domain annotation of the primary structure. Green represents the C-terminal region of FliF from Thermotoga maritima (PDB ID: 5TDY) (40).

We integrated the inner and middle region of the M-ring model with the C34 S-ring model (PDB ID: 6SD4) and constructed the periplasmic structure model of the MS-ring (Fig. 4A to G). The MS-ring is composed of the 34 FliF subunits in the native *Salmonella* basal body. FliF_D1–D2_ adopts two distinct structures in the M-ring. The inner M-ring is formed by 23 copies of FliF_D1–D2_ (Fig. 4A) and is surrounded by 11 copies of FliF_D1–D2_ in a distinct orientation from those in the inner ring (Fig. 4B). These chains form the two conformationally distinct FliF subunit groups, forming the inner and middle region of the M-ring with 23- and 11-fold symmetry, respectively, and are joined to form the S-ring and collar with 34-fold symmetry (Fig. 4C to H). The distance between the C terminus of the S-ring and the density of the outermost region of the M-ring are about 30 Å. Therefore, the 13 residues following the C terminus of the S-ring to the N terminus of FliF TM2 should be adopting an extended conformation.

## DISCUSSION

The crystal structure of RBM1 and RBM2 together with the cryoEM structure of the MS-ring, including a high-resolution structure of the S-ring (RBM3), revealed the periplasmic part of the MS-ring structure. Our cryoEM analysis of the MS-FliG ring complex demonstrated that the MS-ring in the basal body is made up of 34 FliF subunits and is consistent with results of the recent cryoEM studies of the MS-ring formed by full-length FliF and the MS-ring in the flagellar basal body (25). The S-ring with a cylindrical collar is shaped like a boater hat with C34 symmetry, whereas the inner part of the M-ring has a gear wheel-like structure consisting of the core C23 symmetry inner ring and the C11 symmetry cogs surrounding it. Thus, the three RBMs of FliF subunits adopt two distinct arrangements in the M-ring. FliF has two transmembrane helices and a periplasmic region (FliF_P_) between them. FliF_P_ contains three globular RBM domains, namely, FliF_D1_, FliF_D2_, and FliF_D3_, and a long extended up-and-down structure (residues 268 to 382) including two antiparallel β-strands (Fig. 4H; see Fig. S7A in the supplemental material). The N-terminal half of FliF_P_ containing FliF_D1_ and FliF_D2_ adopts the following two distinct domain arrangements in the M-ring: the inner 23 copies form the inner ring and the remaining 11 copies surround the inner ring. The arrangement of FliF_D2_ in the inner ring resembles that of the D2 domain of the SctJ family proteins, such as PrgK and EscJ (Fig. S7C and D). The C-terminal half of FliF_P_ containing FliF_D3_ and the extended structure forms the S-ring and collar. The structure of FliF_D3_ has a common RBM fold and resembles FliF_D2_ (Fig. S7A and C). FliF_D3_ forms a large ring structure with a similar domain arrangement to the inner ring as well as to the SctJ family protein rings (Fig. S7A and D). The long antiparallel β-strands in the extended structure are vertically lined up to form a cylindrical collar with a 68-stranded β-barrel structure. The overall structure of the S-ring resembles the SpoIIIAG structure (Fig. S7A and B), although the number of the subunits and therefore the symmetry are different.

10.1128/mBio.03199-20.7 FIG S7 Structural comparison of various RBM domains. (A to E) The ring (top) and monomeric structures (bottom) of the S-ring (PDB ID: 7CIK) (A), SpoIIIAG (PDB ID: 5WC3) (B), the inner ring of the M-ring (C), PrgK_D2_ (PDB ID: 5TCP) (D), and PrgK_D3_ (left) and the PrgH_D2_ (right) (PDB ID: 5TCP). (F) The monomeric structure of SpoIIIAH (PDB ID: 3TUF). (G) The monomeric structure of SpoIIIAF (PDB ID: 6DCS). Download FIG S7, TIF file, 1.5 MB. Copyright © 2021 Takekawa et al. 2021 Takekawa et al. https://creativecommons.org/licenses/by/4.0/ This content is distributed under the terms of the Creative Commons Attribution 4.0 International license.

Recently, Johnson et al. have solved multiple cryoEM structures of the MS-ring with a variation in subunit stoichiometry from 32 to 35 and found symmetry mismatch between the S-ring and M-ring (24). They mainly described the structure of the MS-ring with C33 symmetry, which our previous work suggests is not the native MS-ring structure in the basal body (25). But even in their model of the MS-ring formed by 34 copies of FliF, the inner M-ring is composed of 22 copies of RBM2 and another set of 10 copies composed of both RBM1 and RMB2 is surrounding it, and therefore, two copies of RBM2 are missing. Although the orientation of each domain in their model is similar to that of our model, the subunit stoichiometries and their azimuthal positions in the inner and middle region of the M-ring are different. Our model contains a complete set of RBM2 with 34 copies, as follows: 23 copies form the inner ring, and 11 copies form the outer surrounding densities of the middle region. Since we built our model based on the cryoEM map of the basal body and the MS-ring formed by 34 copies of full-length FliF, we believe that our model reflects the MS-ring structure of the native flagellum. The MS-FliG ring complex used for our cryoEM analysis of the MS-ring was prepared from the cells expressing not only FliF and FliG but also the export apparatus proteins. Therefore, the export apparatus proteins may be needed for FliF to form the 23- plus 11-subunit structure in the inner part of the M-ring even though they were dissociated from the MS-ring during purification.

The 8.6-Å cryoEM map of the MS-FliG ring complex showed blurred densities in the outer M-ring located below the periphery of the S-ring (Fig. 1C, colored yellow). This density is thought to be formed by the TM2 and C-terminal cytoplasmic region of FliF because the density disappears by truncation of these regions (4). The map also showed another unassigned blurred density below the central hole of the inner M-ring (Fig. 1C, colored gray) where the FlhA nonamer ring is supposed to be located in the basal body, as observed by electron cryotomography (26). However, the MS-FliG ring used to produce the map did not contain any export gate component proteins because they fell off during the purification process. The blurred density is close to the N terminus of FliF_D1_ of the inner M ring model, and the density of FliF_D1_ itself is also relatively poor in the map. Therefore, the N-terminal region of FliF, including TM1 and FliF_D1_ of the inner M-ring, may form a disordered aggregate in the purified MS-FliG ring. The export gate complex might be needed for the proper folding and arrangement of this region.

The flagellar protein export gate complex, which comprises FlhA, FlhB, and the FliP/Q/R complex, is expected to be accommodated in the central hole of the MS-ring (11). The FliP/Q/R complex is needed for the assembly of FlhA into the export gate complex (11). Deletion of the i-loop (residues 161 to 170) surrounding the hole of the inner ring affected the FlhA assembly but not the MS-ring formation (Fig. 3), suggesting that the i-loop interacts with the export gate complex. Recent cryoEM analysis of the injectisome proposed that the SpaP/Q/R complex, which corresponds to the flagellar FliP/Q/R complex, is stuck in the central hole of the SctJ ring, which corresponds to the inner ring of the M-ring (27–29). The SctJ subunits surround the middle of the bullet-shaped P/Q/R complex. In addition, cross-linking experiments have revealed that SctJ directly interacts with the P and R subunits of the complex (27). The inner diameter of the M-ring is comparable to that of the FliP/Q/R complex (27). Therefore, the i-loop of the inner M-ring may directly interact with the FliP/Q/R complex and accommodate it in the central hole. Thus, the FliP/Q/R complex may not be properly held in the inner M-ring without the i-loop, resulting in the collapse of the export gate complex. The inner M-ring shows a 23-fold rotational symmetry, whereas the FliP/Q/R complex is a helical assembly of five FliP, four FliQ, and one FliR molecules. The flexible i-loop would be needed to adjust the inner M-ring interface to the FliP/Q/R complex over the symmetry mismatch.

A conserved A-S(A)-V(I)-X-V(L/I) motif in RBM2 of FliF (ASVFL in residues 172 to 176 of *Aa*-FliF and ASVTV in residues 174 to 178 of *St*-FliF) is believed to be involved in the interaction with FlhA because the nonflagellation phenotype caused by deletion of residues A174 and S175 in *Salmonella* FliF was suppressed by mutations in and around the transmembrane region of FlhA (Fig. S6C) (30). The A-S(A)-V(I)-X-V(L/I) motif is in strand β7 located at the interface to the neighboring subunit in the inner M-ring and is far from the inner membrane in the MS-ring model (Fig. 3A and B), even if the membrane is distorted around the basal body, as depicted in the model of the injectisome basal body (27). Therefore, A174 and S175 do not seem to directly interact with FlhA. The deletion of these two residues may change the size or shape of the central hole of the inner M-ring to cause trouble in accommodating the export gate complex. The suppressor mutations in FlhA may change the conformation of the export gate complex to be fitted into the central hole.

The structure of FliF_D2_ resembles that of FliF_D3_, PrgK_D2_, PrgH_D3_, SpoIIIAF, and SpoIIIAH and the globular domain of SpoIIIAG (Fig. S7). They share an αββαβ motif known as a common RBM. They all form ring structures of different sizes by different numbers of subunits. FliF_D2_ forms a 23-mer ring with an external diameter of about 16 nm. The external diameters of the PrgK 24-mer ring and the FliF_D3_ 34-mer ring are about 17 and 24 nm, respectively. They all share a similar domain orientation and similar neighboring subunit interaction surfaces to form the ring. The RBM domain of SpoIIIAG forms a 30-mer ring with an external diameter of about 21 nm. The PrgH_D3_ ring is composed of 24 subunits, and its external diameter is about 24 nm. Although the domain orientations of these two molecules in their rings are slightly different from that of the FliF_D2_ ring, they also use common interfaces for the neighboring subunit interaction in the ring.

Our structural study has revealed the architecture of the periplasmic region of the MS-ring in the native basal body structure. However, the structure and symmetry of the transmembrane and cytoplasmic regions of the MS-ring remain unclear. The entire structure of the MS-ring is still required to fully understand the mechanism of flagellar assembly and the rotor function.

## MATERIALS AND METHODS

### Bacterial strains and plasmids.

The bacterial strains and plasmids used in this study are listed in Table S2 in the supplemental material. The expression plasmids were constructed by PCR using DNA primers listed in Table S3 in the supplemental material. We cloned the full-length and various fragments of the *fliF* gene from *A. aeolicus* (Fig. S2A). The FliF fragments were designed based on the motif and the secondary structure prediction using PSIPRED (http://bioinf.cs.ucl.ac.uk/psipred/). L121M/L195M mutation in pNT32B and FliF_Δ161–170_ mutation in pNT63 and pNT64 were introduced by the QuikChange site-directed mutagenesis (Agilent, CA, USA). The transformation of E. coli and *Salmonella* was performed by the heat shock method.

10.1128/mBio.03199-20.9 TABLE S2 Strains and plasmids used in this study. Amp^r^, ampicillin resistant; Cm^r^, chloramphenicol resistant; Km^r^, kanamycin resistant; *fliF^Aa^*, *fliF* of *A. aeolicus*; *fliF^St^*, *fliF* of *Salmonella*; *fliF^SA^*, chimeric *fliF*; P*_T7_*, T7 promoter; P*_cspA_*, cold-shock promoter; P*_trc_*, trc promoter. Download Table S2, DOCX file, 0.04 MB. Copyright © 2021 Takekawa et al. 2021 Takekawa et al. https://creativecommons.org/licenses/by/4.0/ This content is distributed under the terms of the Creative Commons Attribution 4.0 International license.

10.1128/mBio.03199-20.10 TABLE S3 DNA primers used in this study. Extra nucleotide sequences for the addition of the restriction enzyme sites, stop codon, sequence on the plasmid vector, and site for the mutation are underlined. Download Table S3, DOCX file, 0.03 MB. Copyright © 2021 Takekawa et al. 2021 Takekawa et al. https://creativecommons.org/licenses/by/4.0/ This content is distributed under the terms of the Creative Commons Attribution 4.0 International license.

### Expression and purification of the *Salmonella* MS-FliG ring complex.

A total of 15 ml of the overnight culture of *Salmonella* SJW1368 (Δ[*cheW-flhD*]) cells harboring pMKM20001 (pTrc99CES/FlhB + FlhA + FliO + FliP + HA-FliQ + FliR-FLAG-His + FliF + FliG) was inoculated into 1.5 liters of fresh 2× YT medium (1.6% [wt/vol] Bacto-tryptone, 1.0% [wt/vol] Bacto-yeast extract, and 0.5% [wt/vol] NaCl) containing 100 μg ml^−1^ ampicillin, and cells were grown at 30°C until the density reached an optical density at 600 nm (OD_600_) of about 0.6. After 30 min of incubation at 4°C, the cells were grown at 16°C for 12 hours. Then, arabinose was added at a final concentration of 0.2% and incubated at 16°C for another 4 hours. Cells were harvested by centrifugation (6,400 × *g*, 10 min, and 4°C) and stored at −80°C. The cells were thawed, resuspended in 55 ml 50 mM Tris-HCl (pH 8.0), 50 mM NaCl, and 5 mM EDTA and disrupted by passage through a French pressure cell (FA-032; Central Scientific Commerce). The cell lysates were centrifuged (20,000 × *g*, 15 min, and 4°C) to remove undisrupted cells. The supernatants were ultracentrifuged (90,000 × *g*, 1 h, and 4°C). The harvested membranes were suspended in 40 ml of 50 mM *N*-cyclohexyl-3-aminopropanesulfonic acid (CAPS)-NaOH (pH 11.0), 50 mM NaCl, 5 mM EDTA, and 0.5% *N*-dodecyl β-d-maltoside (DDM), followed by centrifugation (20,000 × *g*, 20 min, and 4°C), and finally ultracentrifugation (90,000 × *g*, 60 min, and 4°C). Pellets were resuspended in 25 mM Tris-HCl (pH 8.0), 50 mM NaCl, 1 mM EDTA, and 0.1% DDM and incubated at 4°C for 1 hour. The solution was loaded at a 15% to 40% (wt/wt) sucrose density gradient in 25 mM Tris-HCl (pH 8.0), 50 mM NaCl, 1 mM EDTA, and 0.1% DDM. After ultracentrifugation (49,100 × *g*, 13 h, and 4°C), fractions containing FliF and FliG were collected; diluted with 7 volumes of 25 mM Tris-HCl (pH 8.0), 50 mM NaCl, 1 mM EDTA, and 0.1% DDM; and ultracentrifuged (90,000 × *g*, 60 min, and 4°C). Pellets were resuspended in 30 μl of 25 mM Tris-HCl (pH 8.0), 50 mM NaCl, 1 mM EDTA, and 0.1% DDM.

### Sample vitrification and cryoEM data acquisition.

Copper 200 mesh R0.6/1.0 holey carbon grids (Quantifoil) were glow discharged on a glass slide for 30 s. A 2.6-μl aliquot of the sample solution was applied to the grid and blotted by filter paper for 7 s at 100% humidity and 4°C. The grid was frozen by rapid plunging into liquid ethane using a Vitrobot Mark III (Thermo Fisher Scientific, MA, USA). The grids were observed by a Titan Krios FEG transmission electron microscope (Thermo Fisher Scientific) operated at 300 kV with the cryospecimen stage cooled with liquid nitrogen. CryoEM images were recorded with a Falcon II 4k by 4k CMOS direct electron detector (Thermo Fisher Scientific) at a nominal magnification of ×75,000 (image pixel size, 1.07 Å) using the EPU software package. The movie images were collected under a defocus range between 1.0 and 3.0 μm with an exposure time of 2 s at a dose rate of 45 e^−^ pix^−1^ s^−1^ (total accumulated exposure, 90 e^−^ Å^−2^). Each movie image was fractionated into 7 frames.

### Image processing of cryoEM data.

The movie frames were subsequently aligned to compensate for beam-induced motion using MotionCor2 (31), and the parameters for the contrast transfer function (CTF) were estimated using Gctf (32).

A total of 461,944 particle images of the MS-FliG ring complex were automatically picked from 6,961 micrographs using Gautomatch (https://www2.mrc-lmb.cam.ac.uk/research/locally-developed-software/zhang-software/#gauto), and two-dimensional (2D) and three-dimensional (3D) classifications were performed using RELION-2.1 (33) or 3.0 (34).

Particles from good 2D classes were used for making the initial 3D model of the MS-FliG ring complex using cryoSPARC (35). A total of 53,522 particles from the best 3D class were subjected to 3D refinement, which produced a reconstruction with a resolution of 8.6 Å and a B-factor of −578 Å^2^ for the C1 symmetry model and with a resolution of 6.1 Å and a B-factor of −312 Å^2^ for the C11 symmetry model. We did not perform focused classification because the map resolution was not high and the total number of the particle images was not enough.

### Expression and purification of *Aa*-FliF variants.

*E. coli* BL21-CodonPlus(DE3)-RIPL carrying pNT30 to pNT37 was cultured in LB broth containing 50 μg ml^−1^ ampicillin at 37°C to an optical density at 660 nm of 0.6 to 0.8 and cooled on ice for about 30 min. A total of 0.5 mM isopropyl-β-d-thiogalactopyranoside (IPTG) was subsequently added to the culture, and the culture was prolonged for about 20 h at 16°C. Cells were collected by centrifugation (6,700 × *g*) and suspended in Tris-Sodium Chloride (TN) buffer (50 mM Tris-HCl [pH 8.0] and 200 mM NaCl) containing cOmplete, EDTA-free reagent (Roche) and lysozyme (Wako, Japan). The cells were then disrupted by sonication and centrifuged at 20,000 × *g* for 10 min to remove cell debris. The supernatant was ultracentrifuged at 100,000 × *g* for 30 min. All the FliF fragments were successfully expressed (Fig. S2B), and FliF_58–213_, FliF_121–213_, and FliF_230–272,347–396_ fragments were highly soluble (Fig. S2B). The soluble fraction was mixed with Ni-nitrilotriacetic acid (NTA) agarose (Qiagen, Germany) and then incubated on ice for 30 min with gentle mixing. The protein-bound agarose was washed with TN buffer containing 50 mM imidazole, and the proteins were subsequently eluted with TN buffer containing 400 mM imidazole. The protein was concentrated using an Amicon Ultra 10 K device (Merck Millipore, Germany), loaded on a size exclusion column (Superdex 75 10/300 GL; GE Healthcare, UK), and eluted with TN buffer. The peak fraction was collected and concentrated using an Amicon Ultra 10 K device. The expression and purity of the proteins were examined by SDS-PAGE.

The selenomethionine (SeMet) derivative FliF fragment was prepared from E. coli BL21-CodonPlus(DE3) RIL-X carrying pNT32B. The cells were cultured in SeMet minimal medium (0.1% [wt/vol] NH_4_Cl, 0.3% [wt/vol] NH_2_PO_4_, 0.3% [wt/vol] Na_2_HPO_4_, 2% [wt/vol] glucose, 0.03% [wt/vol] MgSO_4_, 0.001% [wt/vol] Fe_2_(SO_4_)_3_, 0.001% [wt/vol] thiamine, and 0.005% [wt/vol] seleno-l-methionine). The proteins were purified in the same way as native FliF fragments.

### Crystallization and X-ray data collection.

Crystallization was carried out using the sitting-drop vapor-diffusion method. Crystallization drops were prepared by mixing 0.5 μl of about 10 to 30 mg ml^−1^ His-FliF_58–213_ with 0.5 μl of the reservoir solution. Initial screening was carried out using the screening kits Wizard classic I and II, cryo I and II (Emerald BioSystems, WA, USA), and crystal screen I and II (Hampton Research, CA, USA); and then the conditions were optimized. Crystals appeared within a week. Because proteins rapidly agglutinate at room temperature, we performed crystallization in a cold room (4°C). The best crystals were grown from the drop prepared by mixing 0.5 μl of 30 mg ml^−1^ protein solution with 0.5 μl of reservoir solution containing 100 mM Na/K phosphate (pH 6.2) and 2.5 M NaCl.

The X-ray diffraction data were collected at synchrotron beamlines BL41XU and BL26B1 in SPring-8 (Harima, Japan) with the approval of the Japan Synchrotron Radiation Research Institute (JASRI) (proposal no. 2016A/B2541 and 2017A/B2588). The crystals were directly transferred into liquid nitrogen for freezing. The diffraction data were collected under nitrogen gas flow at 100 K. The diffraction data were processed with MOSFLM (36) and scaled with AIMLESS (37). The diffraction data statistics are summarized in Table S1. The experimental phase was calculated using the SAD data of the selenomethionine derivative with the program PHENIX (38). The atomic model was built with Coot (39) and refined to 2.3-Å resolution with PHENIX (38) against the native crystal data of His-FliF_58–213_. The refinement statistics are summarized in Table S1.

### *In vivo* disulfide cross-linking.

*Salmonella* SJW1684 cells carrying pITH201 with/without mutations were cultured in LB broth containing 50 mg ml^−1^ ampicillin and 100 μM IPTG at 30°C, and a constant number of cells were collected by centrifugation (13,000 × *g*) and suspended with nonreducing SDS loading buffer, heated at 95°C for 5 min, and subjected to SDS-PAGE followed by immunoblotting using a polyclonal anti-FliF antibody.

### Soft-agar plate assay for motility.

A total of 2 μl of an overnight culture was spotted onto Terrific Broth (TB) soft-agar plates (1% [wt/vol] tryptone, 0.5% [wt/vol] NaCl, 0.25% [wt/vol] agar). The plates were incubated at 30°C for the appropriate time as described in the figure legends. The assay was performed at least three times to confirm the reproducibility of the results.

### Observation of subcellular localization of FliF-GFP and FlhA-YFP with fluorescence microscopy.

The overnight cultures grown in LB broth were inoculated at a 100-fold dilution into TG broth (1% [wt/vol] tryptone, 0.5% [wt/vol] NaCl, and 1% [wt/vol] glycerol) containing 100 μM IPTG and cultured for 4 h at 30°C. Cells were harvested by centrifugation and resuspended in motility medium (10 mM potassium phosphate [pH 7], 0.1 mM EDTA, and 85 mM NaCl). Then, the cells were loaded between a coverslip and a slide glass and incubated for 10 min to be attached to the coverslip surface. Unbound cells were washed away by the motility medium. The cells were observed by a fluorescence microscope (BX53 [Olympus, Japan] for GFP and BX50 [Olympus] for YFP) equipped with a 100-W high-pressure mercury lamp. Images were recorded using a digital charge-coupled-device (CCD) camera (Infinity2-1RM and Infinity Capture [Argo Corporation, Japan] for GFP and ORCA-Flash4.0 and High-speed Recording Software version 1.7.1.0 [Hamamatsu Photonics, Japan] for YFP).

### Data availability.

The atomic coordinate has been deposited in the Protein Data Bank (www.pdb.org) under accession code 7CIK. The cryoEM maps have been deposited in the Electron Microscopy Data Bank under accession codes EMD-30378 and EMD-30379. The coordinate data of the periplasmic region model of the MS-ring are available from the corresponding authors on request.

## References

[1] Berg HC. 2003. The rotary motor of bacterial flagella. Annu Rev Biochem 72:19–54. doi:10.1146/annurev.biochem.72.121801.161737. 12500982

[2] Minamino T, Imada K. 2015. The bacterial flagellar motor and its structural diversity. Trends Microbiol 23:267–274. doi:10.1016/j.tim.2014.12.011. 25613993

[3] Kubori T, Shimamoto N, Yamaguchi S, Namba K, Aizawa SI. 1992. Morphological pathway of flagellar assembly in *Salmonella* Typhimurium. J Mol Biol 226:433–446. doi:10.1016/0022-2836(92)90958-M. 1640458

[4] Ueno T, Oosawa K, Aizawa SI. 1992. M ring, S ring and proximal rod of the flagellar basal body of *Salmonella* Typhimurium are composed of subunits of a single protein, FilF. J Mol Biol 227:672–677. doi:10.1016/0022-2836(92)90216-7. 1404383

[5] Thomas D, Morgan DG, DeRosier DJ. 2001. Structures of bacterial flagellar motors from two FliF-FliG gene fusion mutants. J Bacteriol 183:6404–6412. doi:10.1128/JB.183.21.6404-6412.2001. 11591685 PMC100136

[6] Grünenfelder B, Gehrig S, Jenal U. 2003. Role of the cytoplasmic C terminus of the FliF motor protein in flagellar assembly and rotation. J Bacteriol 185:1624–1633. doi:10.1128/jb.185.5.1624-1633.2003. 12591880 PMC148050

[7] Levenson R, Zhou H, Dahlquist FW. 2012. Structural insights into the interaction between the bacterial flagellar motor proteins FliF and FliG. Biochemistry 51:5052–5060. doi:10.1021/bi3004582. 22670715 PMC3384689

[8] Macnab RM. 2003. How bacteria assemble flagella. Annu Rev Microbiol 57:77–100. doi:10.1146/annurev.micro.57.030502.090832. 12730325

[9] Minamino T. 2014. Protein export through the bacterial flagellar type III export pathway. Biochim Biophys Acta 1843:1642–1648. doi:10.1016/j.bbamcr.2013.09.005. 24064315

[10] Fukumura T, Makino F, Dietsche T, Kinoshita M, Kato T, Wagner S, Namba K, Imada K, Minamino T. 2017. Assembly and stoichiometry of the core structure of the bacterial flagellar type III export gate complex. PLoS Biol 15:e2002281. doi:10.1371/journal.pbio.2002281. 28771466 PMC5542437

[11] McMurry JL, Van Arnam JS, Kihara M, Macnab RM. 2004. Analysis of the cytoplasmic domains of *Salmonella* FlhA and interactions with components of the flagellar export machinery. J Bacteriol 186:7586–7592. doi:10.1128/JB.186.22.7586-7592.2004. 15516571 PMC524908

[12] Morimoto YV, Ito M, Hiraoka KD, Che YS, Bai F, Kami-Ike N, Namba K, Minamino T. 2014. Assembly and stoichiometry of FliF and FlhA in *Salmonella* flagellar basal body. Mol Microbiol 91:1214–1226. doi:10.1111/mmi.12529. 24450479

[13] Hueck CJ. 1998. Type III protein secretion systems in bacterial pathogens of animals and plants. Microbiol Mol Biol Rev 62:379–433. doi:10.1128/MMBR.62.2.379-433.1998. 9618447 PMC98920

[14] Camp AH, Losick R. 2008. A novel pathway of intercellular signalling in *Bacillus subtilis* involves a protein with similarity to a component of type III secretion channels. Mol Microbiol 69:402–417. doi:10.1111/j.1365-2958.2008.06289.x. 18485064 PMC2574792

[15] Bergeron JR. 2016. Structural modeling of the flagellum MS ring protein FliF reveals similarities to the type III secretion system and sporulation complex. PeerJ 4:e1718. doi:10.7717/peerj.1718. 26925337 PMC4768692

[16] Levdikov VM, Blagova EV, McFeat A, Fogg MJ, Wilson KS, Wilkinson AJ. 2012. Structure of components of an intercellular channel complex in sporulating *Bacillus subtilis*. Proc Natl Acad Sci U S A 109:5441–5445. doi:10.1073/pnas.1120087109. 22431604 PMC3325715

[17] Meisner J, Maehigashi T, André I, Dunham CM, Moran CP, Jr. 2012. Structure of the basal components of a bacterial transporter. Proc Natl Acad Sci U S A 109:5446–5451. doi:10.1073/pnas.1120113109. 22431613 PMC3325725

[18] Yip CK, Kimbrough TG, Felise HB, Vuckovic M, Thomas NA, Pfuetzner RA, Frey EA, Finlay BB, Miller SI, Strynadka NCJ. 2005. Structural characterization of the molecular platform for type III secretion system assembly. Nature 435:702–707. doi:10.1038/nature03554. 15931226

[19] Schraidt O, Marlovits TC. 2011. Three-dimensional model of *Salmonella*’s needle complex at subnanometer resolution. Science 331:1192–1195. doi:10.1126/science.1199358. 21385715

[20] Bergeron JRC, Worrall LJ, De S, Sgourakis NG, Cheung AH, Lameignere E, Okon M, Wasney GA, Baker D, McIntosh LP, Strynadka NCJ. 2015. The modular structure of the inner-membrane ring component PrgK facilitates assembly of the type III secretion system basal body. Structure 23:161–172. doi:10.1016/j.str.2014.10.021. 25533490

[21] Worrall LJ, Hong C, Vuckovic M, Deng W, Bergeron JRC, Majewski DD, Huang RK, Spreter T, Finlay BB, Yu Z, Strynadka NCJ. 2016. Near-atomic-resolution cryo-EM analysis of the *Salmonella* T3S injectisome basal body. Nature 540:597–601. doi:10.1038/nature20576. 27974800

[22] Zeytuni N, Flanagan KA, Worrall LJ, Massoni SC, Camp AH, Strynadka NCJ. 2018. Structural and biochemical characterization of SpoIIIAF, a component of a sporulation-essential channel in *Bacillus subtilis*. J Struct Biol 204:1–8. doi:10.1016/j.jsb.2018.06.002. 29886194

[23] Zeytuni N, Hong C, Flanagan KA, Worrall LJ, Theiltges KA, Vuckovic M, Huang RK, Massoni SC, Camp AH, Yu Z, Strynadka NCJ. 2017. Near-atomic resolution cryoelectron microscopy structure of the 30-fold homooligomeric SpoIIIAG channel essential to spore formation in *Bacillus subtilis*. Proc Natl Acad Sci U S A 114:E7073–E7081. doi:10.1073/pnas.1704310114. 28784753 PMC5576796

[24] Johnson S, Fong YH, Deme JC, Furlong EJ, Kuhlen L, Lea SM. 2020. Symmetry mismatch in the MS-ring of the bacterial flagellar rotor explains the structural coordination of secretion and rotation. Nat Microbiol 5:966–975. doi:10.1038/s41564-020-0703-3. 32284565 PMC7320910

[25] Kawamoto A, Miyata T, Makino F, Kinoshita M, Minamino T, Imada K, Kato T, Namba K. 2020. Native structure of flagellar MS ring is formed by 34 subunits with 23-fold and 11-fold subsymmetries. biorXiv doi:10.1101/2020.10.11.334888. PMC8270960 34244518

[26] Kawamoto A, Morimoto YV, Miyata T, Minamino T, Hughes KT, Kato T, Namba K. 2013. Common and distinct structural features of *Salmonella* injectisome and flagellar basal body. Sci Rep 3:3369. doi:10.1038/srep03369. 24284544 PMC3842551

[27] Kuhlen L, Abrusci P, Johnson S, Gault J, Deme J, Caesar J, Dietsche T, Mebrhatu MT, Ganief T, Macek B, Wagner S, Robinson CV, Lea SM. 2018. Structure of the core of the type III secretion system export apparatus. Nat Struct Mol Biol 25:583–590. doi:10.1038/s41594-018-0086-9. 29967543 PMC6233869

[28] Johnson S, Kuhlen L, Deme JC, Abrusci P, Lea SM. 2019. The structure of an injectisome export gate demonstrates conservation of architecture in the core export gate between flagellar and virulence type III secretion systems. mBio 10:e00818-19. doi:10.1128/mBio.00818-19. 31239376 PMC6593402

[29] Hu J, Worrall LJ, Vuckovic M, Hong C, Deng W, Atkinson CE, Brett Finlay B, Yu Z, Strynadka NCJ. 2019. T3S injectisome needle complex structures in four distinct states reveal the basis of membrane coupling and assembly. Nat Microbiol 4:2010–2019. doi:10.1038/s41564-019-0545-z. 31427728

[30] Kihara M, Minamino T, Yamaguchi S, Macnab RM. 2001. Intergenic suppression between the flagellar MS ring protein FliF of *Salmonella* and FlhA, a membrane component of its export apparatus. J Bacteriol 183:1655–1662. doi:10.1128/JB.183.5.1655-1662.2001. 11160096 PMC95050

[31] Zheng SQ, Palovcak E, Armache JP, Verba KA, Cheng Y, Agard DA. 2017. MotionCor2: anisotropic correction of beam-induced motion for improved cryo-electron microscopy. Nat Methods 14:331–332. doi:10.1038/nmeth.4193. 28250466 PMC5494038

[32] Zhang K. 2016. Gctf: real-time CTF determination and correction. J Struct Biol 193:1–12. doi:10.1016/j.jsb.2015.11.003. 26592709 PMC4711343

[33] Kimanius D, Forsberg BO, Scheres SH, Lindahl E. 2016. Accelerated cryo-EM structure determination with parallelisation using GPUS in RELION-2. Elife 5:e18722. doi:10.7554/eLife.18722. 27845625 PMC5310839

[34] Zivanov J, Nakane T, Forsberg BO, Kimanius D, Hagen WJ, Lindahl E, Scheres SH. 2018. New tools for automated high-resolution cryo-EM structure determination in RELION-3. Elife 7:e42166. doi:10.7554/eLife.42166. 30412051 PMC6250425

[35] Punjani A, Rubinstein JL, Fleet DJ, Brubaker MA. 2017. CryoSPARC: algorithms for rapid unsupervised cryo-EM structure determination. Nat Methods 14:290–296. doi:10.1038/nmeth.4169. 28165473

[36] Battye TGG, Kontogiannis L, Johnson O, Powell HR, Leslie AGW. 2011. iMOSFLM: a new graphical interface for diffraction-image processing with MOSFLM. Acta Crystallogr D Biol Crystallogr 67:271–281. doi:10.1107/S0907444910048675. 21460445 PMC3069742

[37] Evans PR, Murshudov GN. 2013. How good are my data and what is the resolution? Acta Crystallogr D Biol Crystallogr 69:1204–1214. doi:10.1107/S0907444913000061. 23793146 PMC3689523

[38] Adams PD, Afonine PV, Bunkóczi G, Chen VB, Davis IW, Echols N, Headd JJ, Hung LW, Kapral GJ, Grosse-Kunstleve RW, McCoy AJ, Moriarty NW, Oeffner R, Read RJ, Richardson DC, Richardson JS, Terwilliger TC, Zwart PH. 2010. PHENIX: a comprehensive Python-based system for macromolecular structure solution. Acta Crystallogr D Biol Crystallogr 66:213–221. doi:10.1107/S0907444909052925. 20124702 PMC2815670

[39] Emsley P, Lohkamp B, Scott WG, Cowtan K. 2010. Features and development of Coot. Acta Crystallogr D Biol Crystallogr 66:486–501. doi:10.1107/S0907444910007493. 20383002 PMC2852313

[40] Lynch MJ, Levenson R, Kim EA, Sircar R, Blair DF, Dahlquist FW, Crane BR. 2017. Co-folding of a FliF-FliG split domain forms the basis of the MS:C ring interface within the bacterial flagellar motor. Structure 25:317–328. doi:10.1016/j.str.2016.12.006. 28089452 PMC5387689

[41] Hu B, Lara-Tejero M, Kong W, Galán JE, Liu J. 2017. In situ molecular architecture of the salmonella type III secretion machine. Cell 168:1065–1074. doi:10.1016/j.cell.2017.02.022. 28283062 PMC5393631

[42] Nivón LG, Moretti R, Baker D. 2013. A pareto-optimal refinement method for protein design scaffolds. PLoS One 8:e59004. doi:10.1371/journal.pone.0059004. 23565140 PMC3614904

