# Supplementary figures and images for: Two Distinct Conformations in 34 FliF Subunits Generate Three Different Symmetries within the Flagellar MS-Ring
Source: mBio. 2021 Mar 2;12(2):e03199-20. doi: 10.1128/mBio.03199-20 (PMC8092281; doi:10.1128/mBio.03199-20)

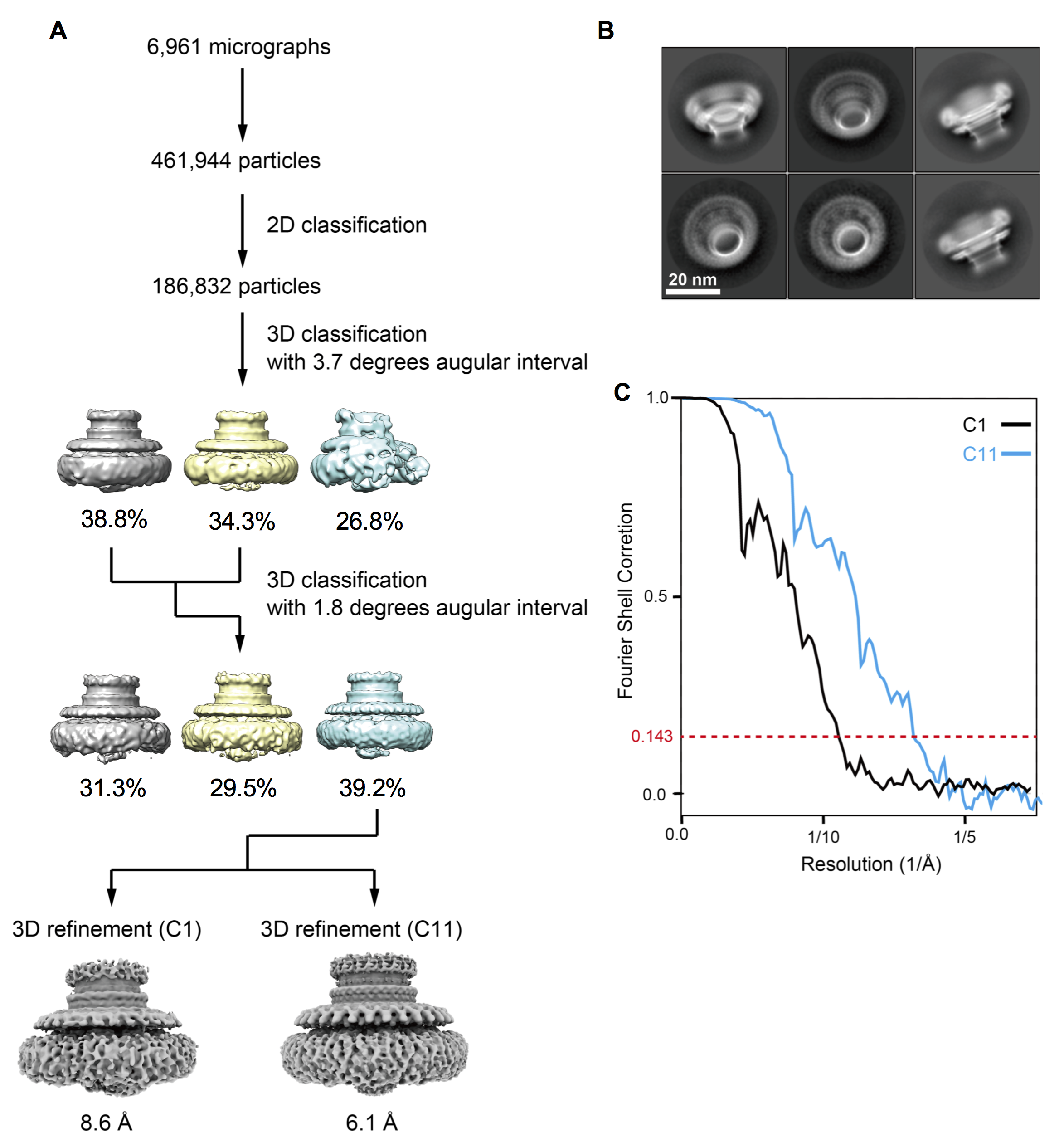

Supplement: FIG S1 [file mBio.03199-20-sf001.tif]

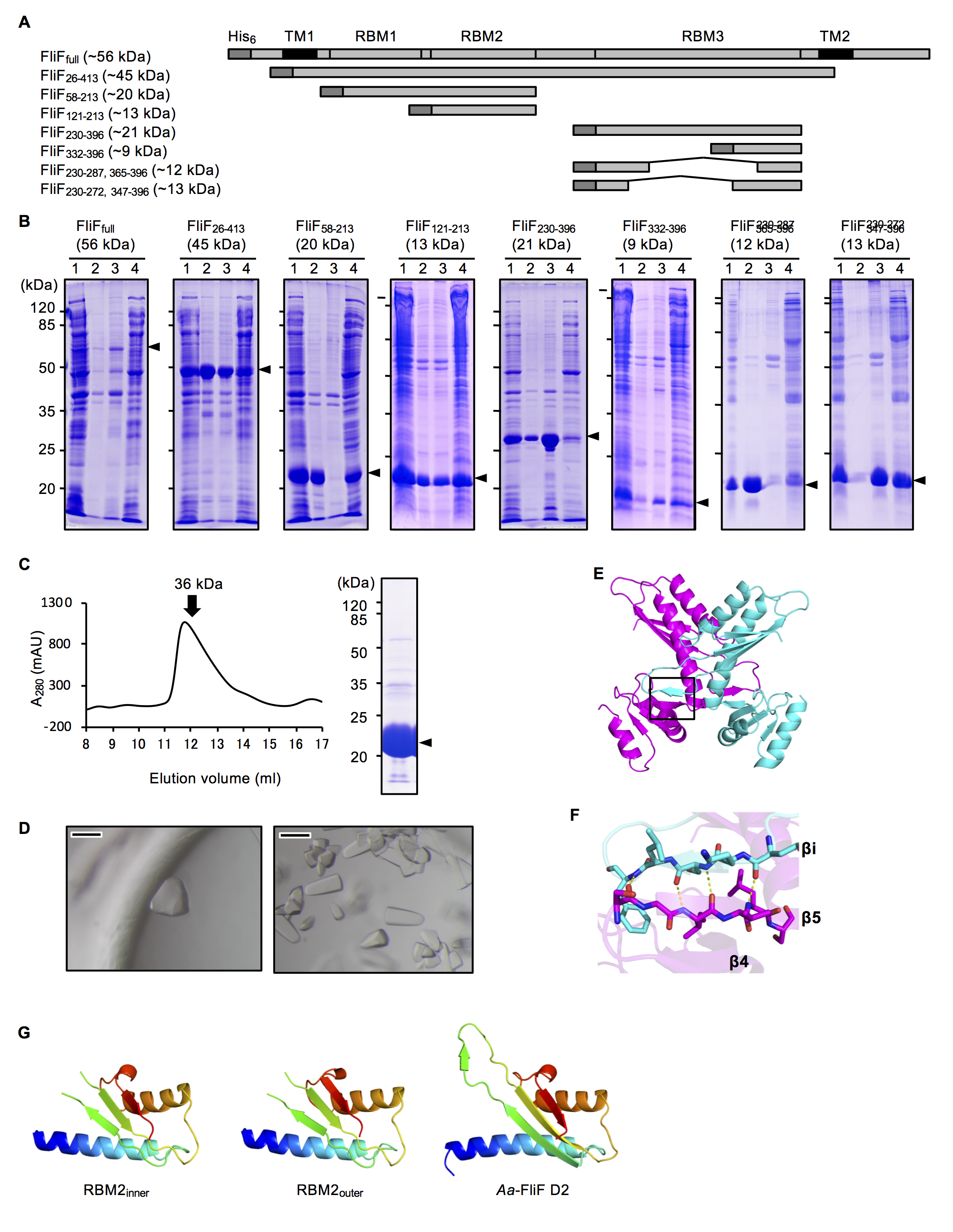

Supplement: FIG S2 [file mBio.03199-20-sf002.tif]

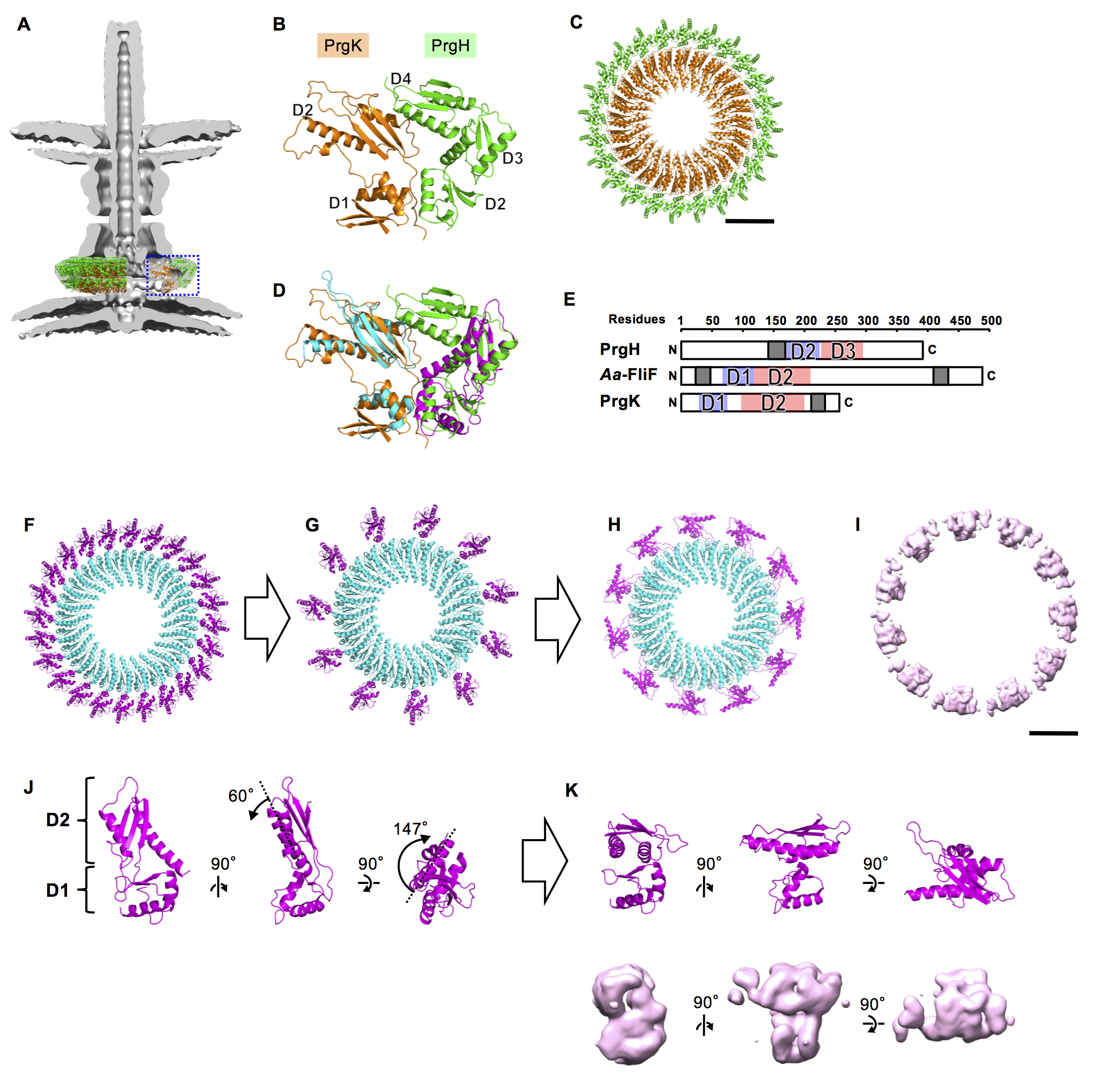

Supplement: FIG S3 [file mBio.03199-20-sf003.tif]

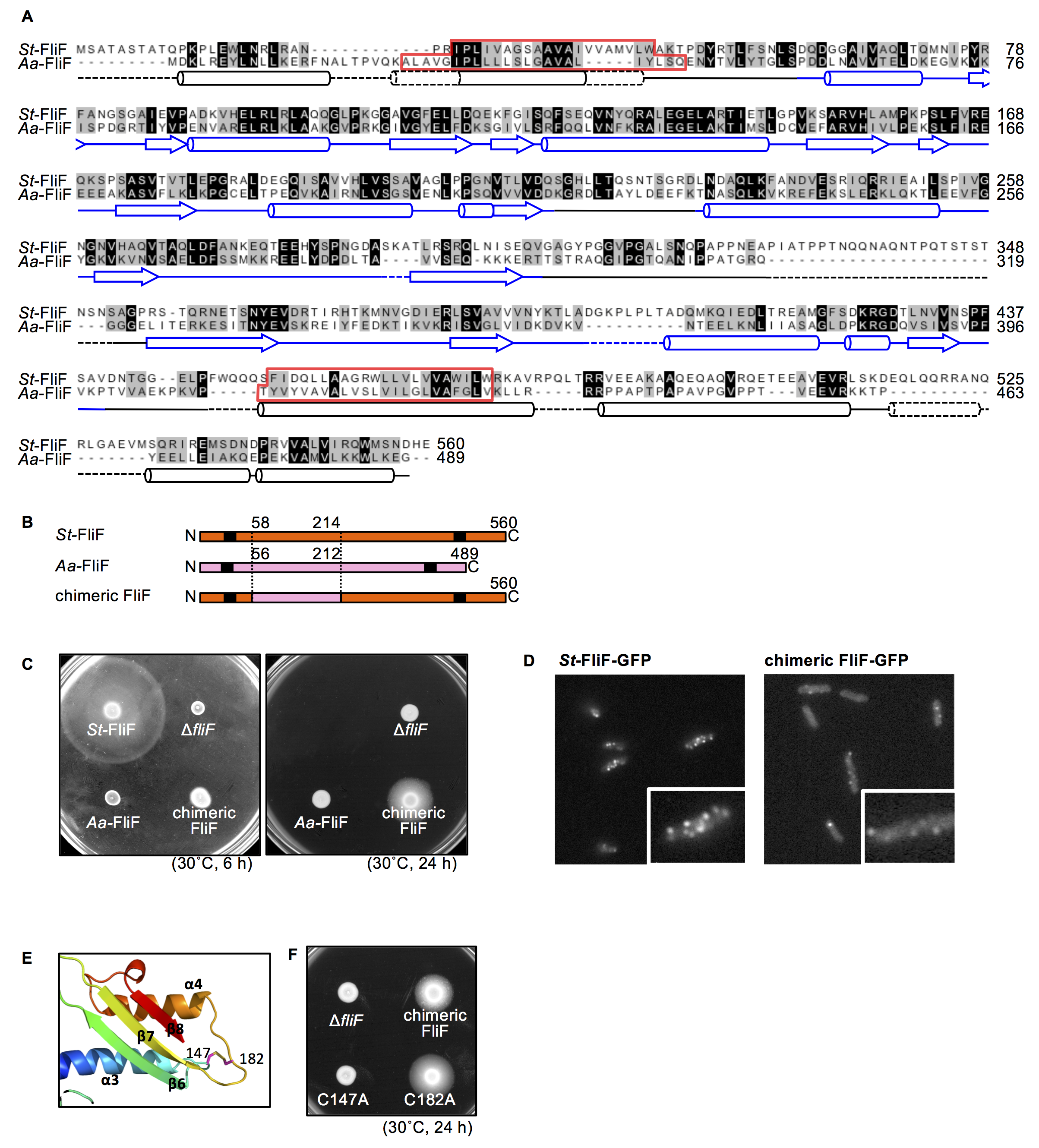

Supplement: FIG S4 [file mBio.03199-20-sf004.tif]

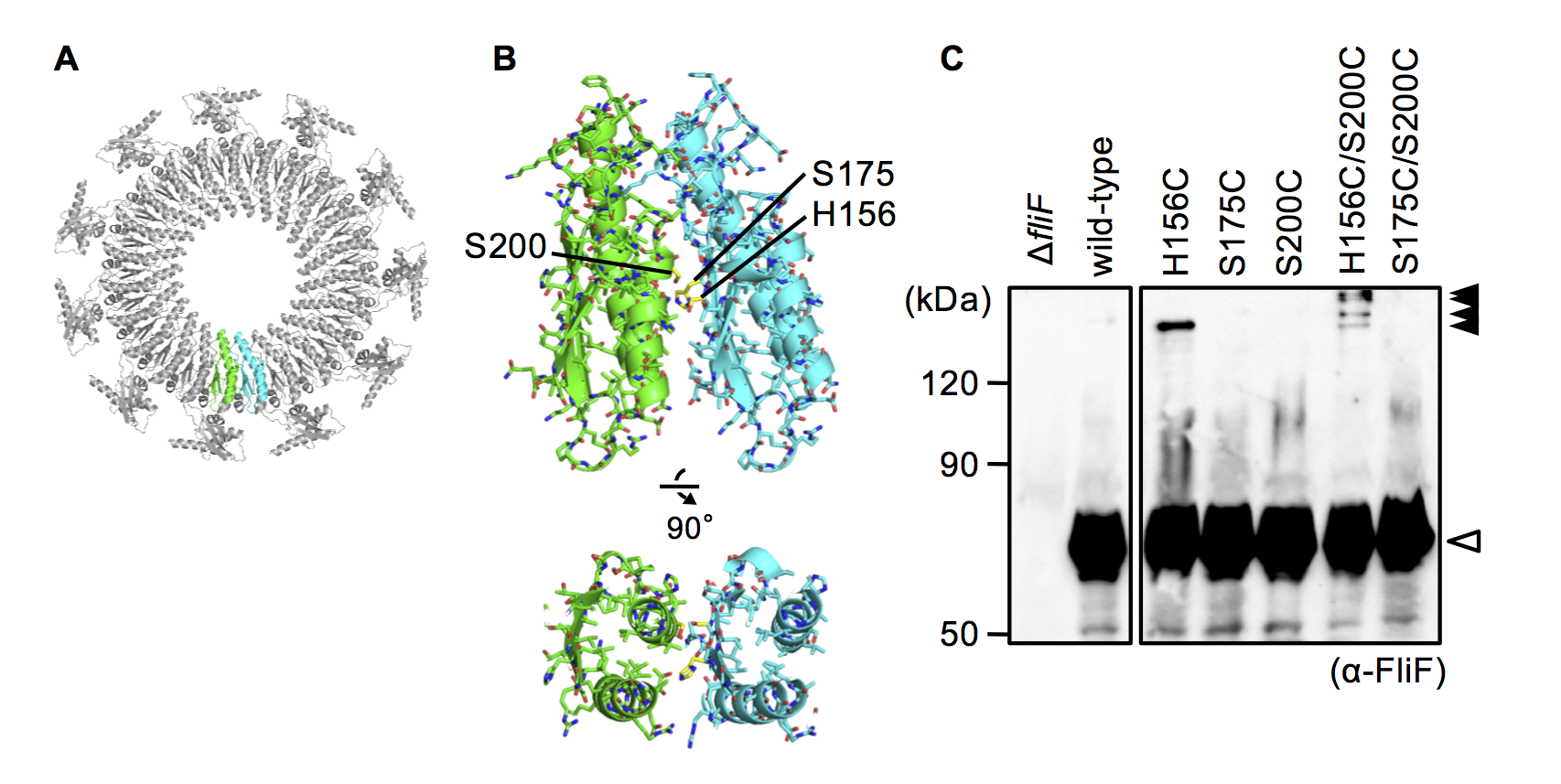

Supplement: FIG S5 [file mBio.03199-20-sf005.tif]

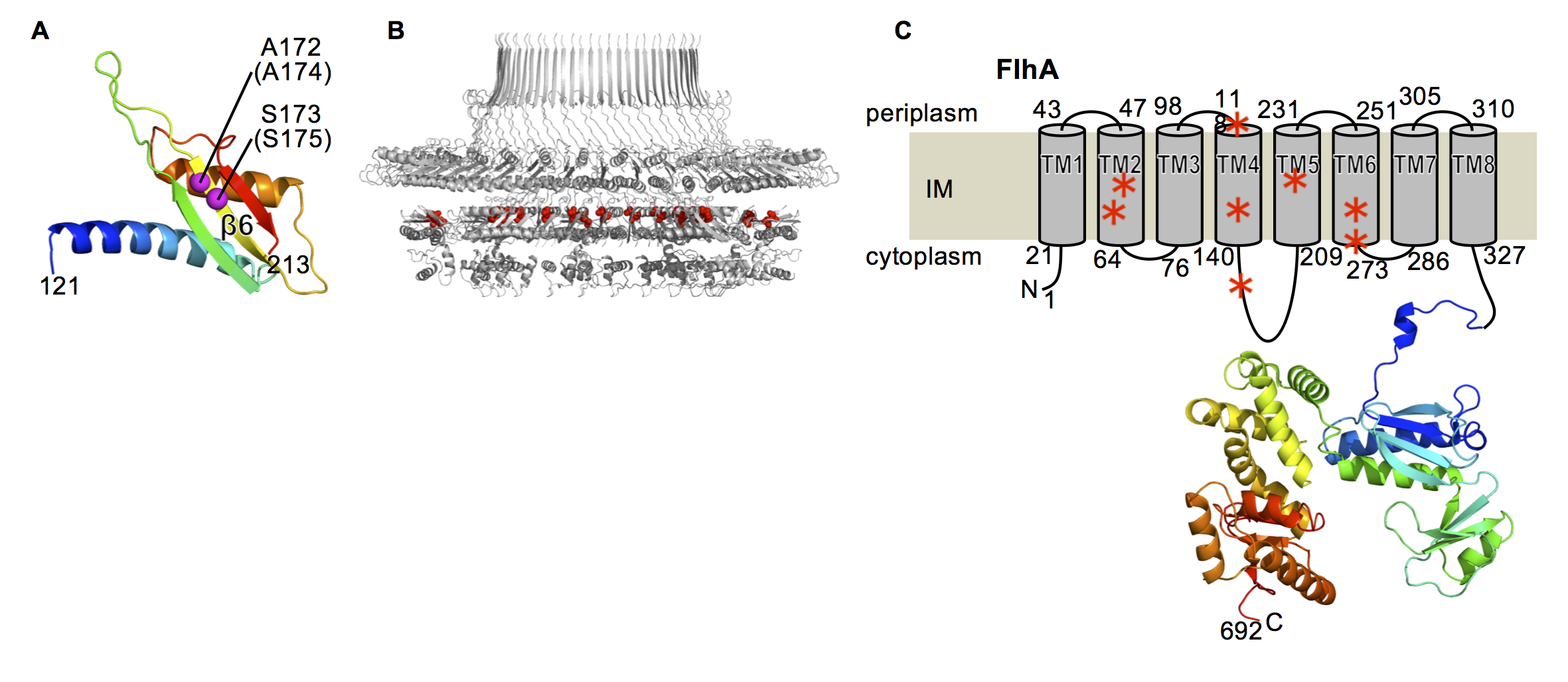

Supplement: FIG S6 [file mBio.03199-20-sf006.tif]

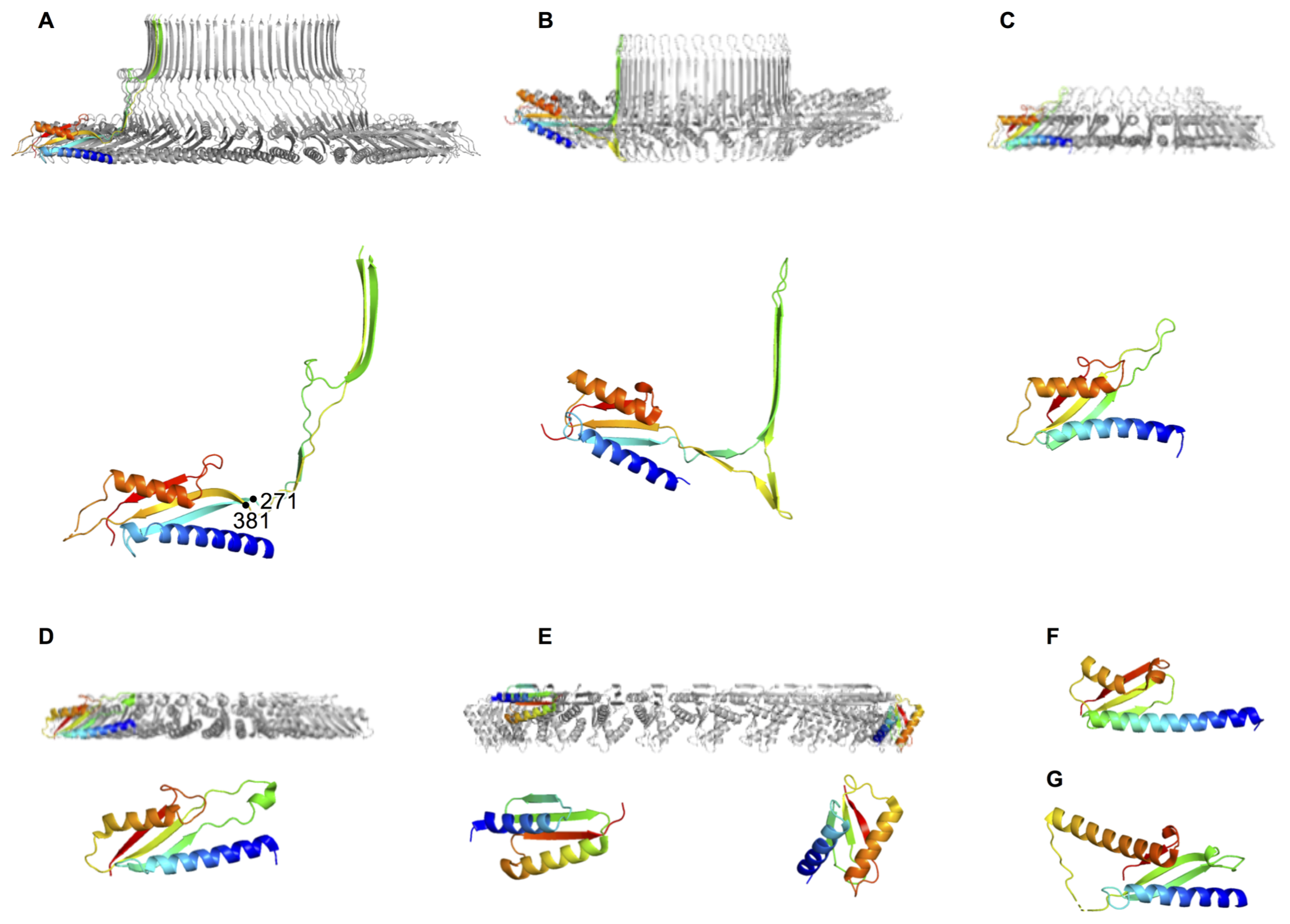

Supplement: FIG S7 [file mBio.03199-20-sf007.tif]
