# Supplementary material for: Two Distinct Conformations in 34 FliF Subunits Generate Three Different Symmetries within the Flagellar MS-Ring
Source: mBio. 2021 Mar 2;12(2):e03199-20. doi: 10.1128/mBio.03199-20 (PMC8092281; doi:10.1128/mBio.03199-20)
Supplement: TABLE S1 [file mBio.03199-20-st001.docx]

Table S1. Summary of the X-ray data statistics.

| Diffraction data statistics |  |  |
| --- | --- | --- |
|  | *Aa*-FliF_58-213_ (native) | *Aa*-FliF_58-213_ (L121M, L195M, selenomethionine derivative) |
| Space group | *H*3 | *H*3 |
| Cell dimensions (Å) | *a* = *b* = 121.75, *c* = 71.72 | *a* = *b* = 120.33, *c* = 72.30 |
| Wave length (Å) | 1.0000 | 0.9792 |
| Resolution range (Å) | 60.9–2.3 (2.4–2.3) | 60.2–2.7 (2.8–2.7) |
| Number of observations | 55,331 (5,675) | 57,677 (7,778) |
| Number of unique reflections | 17,374 (1,750) | 10,715 (1,440) |
| Completeness (%) | 96.6 (98.6) | 99.9 (99.9) |
| Multiplicity | 3.2 (3.2) | 5.4 (5.4) |
| I/σ (*I*) | 9.8 (2.9) | 18.3 (4.1) |
| R_merge_ (%) | 5.4 (30.5) | 7.7 (38.6) |
| Refinement statistics |  |  |
| Resolution (Å) | 60.9–2.3 (2.4–2.3) |  |
| Number of reflections working | 55,331 (5,675) |  |
| Number of reflections test | 17,374 (1,750) |  |
| R_w_ (%) | 20.8 (27.6) |  |
| R_free_ (%) | 25.6 (34.2) |  |
| Root mean square deviation |  |  |
| Bond length (Å) | 0.002 |  |
| Bond angle (°) | 0.629 |  |
| B factors |  |  |
| Protein atoms | 68.1 |  |
| ligand atoms | 64.1 |  |
| Solvent atoms | 52.4 |  |
| Ramachandran plot (%) |  |  |
| Most favored allowed | 94.2 |  |
| Additionally allowed | 5.5 |  |
| Generously allowed | 0.3 |  |
| Disallowed | 0.0 |  |
| Number of protein atoms | 2,444 |  |
| Number of ligand atoms | 8 |  |
| Number of solvent atoms | 156 |  |

The values in parentheses are for the highest-resolution shell.

R_w_= Σ || Fo | - | Fc || / Σ | Fo |, R_free_= Σ || Fo | - | Fc || / Σ | Fo |
