# Supplementary material for: Two Distinct Conformations in 34 FliF Subunits Generate Three Different Symmetries within the Flagellar MS-Ring
Source: mBio. 2021 Mar 2;12(2):e03199-20. doi: 10.1128/mBio.03199-20 (PMC8092281; doi:10.1128/mBio.03199-20)
Supplement: TABLE S2 [file mBio.03199-20-st002.docx]

Table S2. Strains and plasmids used in this study.

| Strain or plasmid | Description | Source or reference |
| --- | --- | --- |
| *E. coli* |  |  |
| DH5α | F^-^ λ^-^ *recA1 hsdR17 endA1 supE44 thi-1 relA1 gyrA96*Δ(*argF*-*lacZYA*) U169 φ80d*lacZ*ΔM15)  (Recipient for cloning experiments) | (1) |
| BL21-CodonPlus(DE3)-RIPL | F^-^ *ompT hsdS*(r_B_^-^ m_B_^-^) *dcm*^+^ Tet^r^ *gal* λ(DE3) *endA* Hte [*argU proL* Cm^r^] [*argU ileY* *leuW* Strep/Spec^r^]  (Host for expression of genes from *A. aeolicus*) | Agilent |
| BL21-CodonPlus(DE3)-RIL-X | F^–^ *ompT hsdS*(r_B_^–^ m_B_^–^) *dcm*^+^ Tet^r^ gal λ(DE3) *endA* Hte *metA::Tn5*(KanS^r^) [*argU ileY leuW Cm^r^*]  (Host for production of selenomethionine derivative of protein) | Agilent |
| *Salmonella* |  |  |
| SJW1103 | Wild-type for flagellar motility and chemotaxis | (2) |
| SJW1684 | SJW1103Δ*fliF* | (3) |
| YVMA002 | SJW1103Δ*fliF*Δ*flhA::tetRA* | (4) |
| JR501 | For converting plasmid to *Salmonella* compatibility | (5) |
| SJW1368 | ∆(*cheW-flhD*); master operon mutant | (6) |
| Plasmids |  |  |
| pET-3b | Amp^r^ P*_T7_* (Expression vector) | Novagen |
| pKOT105 | pET-3b- *fliF^St^* | (7) |
| pColdI | Amp^r^ P*_cspA_* (Cold shock expression vector) | Takara |
| pTrc99CES | Amp^r^ P*_trc_* (Expression vector) | (8) |
| pMKM20001 | pTrc99CES/ FlhB + FlhA + FliO + FliP + HA-FliQ + FliR-FLAG-His + FliF + FliG | This study |
| pNT50 | pColdI- *fliF^Aa^* | This study |
| pNT51 | pColdI- *fliF^Aa^_26–413_* | This study |
| pNT52A | pColdI- *fliF^Aa^_58–213_* | This study |
| pNT52B | pColdI- *fliF^Aa^_58–213_(L121M/L195M)* | This study |
| pNT53 | pColdI- *fliF^Aa^_121–213_* | This study |
| pNT54 | pColdI- *fliF^Aa^_230–396_* | This study |
| pNT55 | pColdI- *fliF^Aa^_332–396_* | This study |
| pNT56 | pColdI- *fliF^Aa^_230–287, 365–396_* | This study |
| pNT57 | pColdI- *fliF^Aa^_230–272, 347–396_* | This study |
| pSBETa | Km^r^ P*_T7_* *argU* | (9) |
| pNT58 | pSBETa- *fliF^St^* | This study |
| pNT59 | pSBETa- *fliF^Aa^* | This study |
| pNT60 | pSBETa- *fliF^SA^* | This study |
| pNT61 | pSBETa- *fliF^St^-egfp* | This study |
| pNT62 | pSBETa- *fliF^SA^-egfp* | This study |
| pNT63 | pSBETa- *fliF^St^_Δ161–170_* | This study |
| pNT64 | pSBETa- *fliF^St^_Δ161–170_-egfp* | This study |
| pTrc99A | Amp^r^ P*_trc_* | Addgene |
| pITH201 | pTrc99A *fliFG*^St^ | Hiroyuki Terashima |
| pET19b | Amp^r^ P*_T7_* | Novagen |
| pYVM054 | pET19b- *his-flhA-eyfp^A206K^* | (4) |

Amp^r^, ampicillin resistant; Cm^r^, chloramphenicol resistant; Km^r^, kanamycin resistant; *fliF^Aa^*, *fliF* of *A. aeolicus*; *fliF^St^*, *fliF* of *Salmonella*; *fliF^SA^*, chimeric *fliF*; P*_T7_*, T7 promoter; P*_cspA_*, cold-shock promoter; P*_trc_*, trc promoter.

References

1. S. G. N. Grant, J. Jessee, F. R. Bloom, D. Hanahan, Proc Natl Acad Sci USA 87:4645–4649, 1990.
2. S. Yamaguchi, H. Fujita, K. Sugata, T. Taira, T. Iino, J Gen Microbiol 130:255–265, 1984.
3. T. Kubori, N. Shimamoto, S. Yamaguchi, K. Namba, S. I. Aizawa, J Mol Biol 226:433–446, 1992.
4. Y. V. Morimoto, *et al.*, Mol Microbiol 91:1214–1226, 2014.
5. J. Ryu, R. J. Hartin, Biotechniques 8:43–45, 1990.
6. K. Ohnishi, Y. Ohto, SI. Aizawa, R. M. Macnab, T. Iino, J Bacteriol 176:2272-2281, 1994.
7. T Ueno, K Oosawa, SI Aizawa, J Mol Biol 227: 672–677, 1992.
8. T Fukumura, F Makino, T Dietsche, M Kinoshita, T Kato, S Wagner, K Namba, K Imada, T Minamino. PLoS Biol 15:e2002281, 2017.
9. P. M. Schenk, S. Baumann, R. Mattes, H. H. Steinbiss, Biotechniques 19:196–198, 1995.
