# Supplementary material for: Two Distinct Conformations in 34 FliF Subunits Generate Three Different Symmetries within the Flagellar MS-Ring
Source: mBio. 2021 Mar 2;12(2):e03199-20. doi: 10.1128/mBio.03199-20 (PMC8092281; doi:10.1128/mBio.03199-20)
Supplement: TABLE S3 [file mBio.03199-20-st003.docx]

Table S3. DNA primers used in this study.

| Primer name | Sequence (5’ to 3’) | Accession number and corresponding sequence (nt) |
| --- | --- | --- |
| NdeI-*AafliF*(+) | GGAATTCCATATGGACAAGTTAAGGGAATAC | NC_000918 (832044–832064; complement) |
| *AafliF*-XbaI(-) | GCTCTAGATCAGCCCTCTTTTAACCAC | NC_000918 (830595–830613) |
| NdeI-*AafliF*(A26)(+) | GGAATTCCATATGGCGGTAGGCATTCCTCTTTTG | NC_000918 (831969–831989; complement) |
| *AafliF*(Y413)-XbaI(-) | GCTCTAGATCAGTAAACGTAGGTAGGAACTTTG | NC_000918 (830826–830847) |
| NdeI-*AafliF*(P58)(+) | GGAATTCCATATGCCGGATGACCTGAACGCTG | NC_000918 (831875–831893; complement) |
| NdeI-*AafliF*(L121)(+) | GGAATTCCATATGCTTTCCCGCTTTCAACAAC | NC_000918 (831686–831704; complement) |
| *AafliF*(D213)-XbaI(-) | GCTCTAGATCAGTCGTCTACCACTACCACC | NC_000918 (831426–831444) |
| NdeI-*AafliF*(A230)(+) | GGAATTCCATATGGCCTCTCAATTAAAAGTAAAAAG | NC_000918 (831355–831377; complement) |
| NdeI-*AafliF*(I332)(+) | GGAATTCCATATGATAACCAATTACGAGGTGAG | NC_000918 (831052–831071; complement) |
| *AafliF*(F396)-XbaI(-) | GCTCTAGATCAAAAAGGAACGCTAACGATAG | NC_000918 (830877–830896) |
| NdeI-*AafliF*(V455)(+) | GGAATTCCATATGGTAGAAGAAGTCAGAAAGAAG | NC_000918 (830682–830702; complement) |
| *AafliF*-L176M (+) | GGCCTCCGTTTTTATGAAGTTAAAACCGGG | NC_000918 (831523–831552; complement) |
| *AafliF*-L176M (-) | CCCGGTTTTAACTTCATAAAAACGGAGGCC | NC_000918 (831523–831552) |
| *AafliF*-L195M(+) | CTATAAGAAATATGGTGTCTGGGAG | NC_000918 (831469–831493; complement) |
| *AafliF*-L195M(-) | CTCCCAGACACCATATTTCTTATAG | NC_000918 (831469–831493) |
| NdeI-*StfliF*(+) | GGAATTCCATATGAGTGCGACTGCATCGAC | NC_003197 (2056536–2056555) |
| StfliF-*BamHI*(-) | CGGGATCCTTACTCATGATCGTTACTCATC | NC_003197 (2058197–2058218; complement) |
| AafliF-*BamHI*(-) | CGGGATCCTCAGCCCTCTTTTAACCACTTTTTC | NC_000918 (830595–830619) |
| *fliF*-chimera-upper(+) | GCTATTTAGCAACCTTTCTCCGGATGACCTGAAC | NC_003197 (2056694–2056706), NC_000918 (831879–831899; complement) |
| *fliF*-chimera-upper(-) | GTTCAGGTCATCCGGAGAAAGGTTGCTAAATAGC | NC_003197 (2056694–2056706 complement), NC_000918 (831879–831899) |
| *fliF*-chimera-lower(+) | GCAGGTGGTAGTGGTAGATCAATCCGGTCATCTG | NC_000918 (831430–831447; complement), NC_003197 (2057177–2057191) |
| *fliF*-chimera-lower(-) | CAGATGACCGGATTGATCTACCACTACCACCTGC | NC_000918 (831430–831447), NC_003197 (2057177–2057191; complement) |
| *StfliF*-gfp(+) | CGATCATGAGGGTGGCGTGAGCAAGGGCGAGGAG | NC_003197 (2058206–2058215), U76561 (292–309) |
| *StfliF*-gfp(-) | CACGCCACCCTCATGATCGTTACTCATCCACTGG | U76561 (292–294, complement), NC_003197 (2058191–2058215; complement) |
| *gfp*-HindIII-BamHI(-) | CGGGATCCAAGCTTACTTGTACAGCTCGTCCATGC | U76561 (986–1008, complement) |
| *StfliF*Δ161-170(+) | CCTGGCGATGCCATCCCCTTCCGCCTCCGTCACCG | NC_003197 (2057003–2057015, 2057046–2057067) |
| *StfliF*Δ161-170(-) | AGGCGGAAGGGGATGGCATCGCCAGGTGAACGCGG | NC_003197 (2056994–2057015, 2057046–2057058, complement) |

Extra nucleotide sequences for the addition of the restriction enzyme sites, stop codon, sequence on the plasmid vector, and site for the mutation are underlined.
